# Structures and Properties of Naturally Occurring Polyether Antibiotics

**DOI:** 10.1155/2013/162513

**Published:** 2013-03-17

**Authors:** Jacek Rutkowski, Bogumil Brzezinski

**Affiliations:** Department of Biochemistry, Faculty of Chemistry, Adam Mickiewicz University, Grunwaldzka 6, 60-780 Poznań, Poland

## Abstract

Polyether ionophores represent a large group of natural, biologically active substances produced by *Streptomyces spp*. They are lipid soluble and able to transport metal cations across cell membranes. Several of polyether ionophores are widely used as growth promoters in veterinary. Polyether antibiotics show a broad spectrum of bioactivity ranging from antibacterial, antifungal, antiparasitic, antiviral, and tumour cell cytotoxicity. Recently, it has been shown that some of these compounds are able to selectively kill cancer stem cells and multidrug-resistant cancer cells. Thus, they are recognized as new potential anticancer drugs. The biological activity of polyether ionophores is strictly connected with their molecular structure; therefore, the purpose of this paper is to present an overview of their formula, molecular structure, and properties.

## 1. Introduction

Polyether ionophores are very large and important group of naturally occurring compounds. Increased interest in this type of compound has been observed in recent years. There are over 120 naturally occurring ionophores known [1]. Major commercial use of the ionophores is to control coccidiosis. They are also used as growth promoters in ruminants. These compounds specifically target the ruminal bacterial population increasing production efficiency. In 2003, the antimicrobials most commonly used in beef cattle production were ionophores. Lasalocid (Avatec, Bovatec), monensin (Coban, Rumensin, and Coxidin), salinomycin (Bio-cox, Sacox), narasin (Monteban, Maxiban), maduramycin (Cygro), and laidlomycin propionate (Cattlyst) were the ionophores of combined yearly sales of more than $150 million [2]. Ionophores can also be used in the production of ion-selective electrodes [3, 4].

All applications of ionophores mentioned above are closely related to their ability to form complexes with metal cations (host-guest complexes) and transport these complexes across lipid bilayers and cell membranes. According to the results of X-ray studies, the metal cation sites in a cage formed by oxygen atoms of the ionophore. The polyether antibiotics form neutral complexes with monovalent cations (i.e., monensin, salinomycin) or divalent metal cations (i.e., lasalocid, calcimycin) as well as with organic bases (i.e., lasalocid). The neutral complexes with cations are formed more preferably than charged complexes because the carboxylic group is deprotonated at physiological pH. However, recently it has been shown that polyether ionophores can also act as neutral ionophores and transport cations as charged complexes. Typically, the complexation of the cation is connected with formation of a pseudocyclic structure which is stabilized by intramolecular hydrogen bonds formed between the carboxylic group and the hydroxyl groups. The ring of ionophore molecule is wrapped around this hydrophilic cage rendering the whole complex lipophilic. The mechanism of transport of a cation by polyether ionophores is attributed to their ability to exchange protons and cations in an electroneutral process. In this type of transport of the cations (M^+^), the polyether ionophore anion (I–COO^−^) binds the metal cation or proton (H^+^) to give a neutral salt (I–COO^−^M^+^) or a neutral polyether ionophore in acidic form (I–COOH), respectively, and only uncharged molecules containing either the metal cation or proton can move through the cell membrane. The whole process leads to changes in Na^+^/K^+^ gradient and to an increase in the osmotic pressure inside the cell, causing swelling and vacuolization, and finally death of the bacteria cell. The remarkable selectivity of some ionophores is attributed to the size of the cage. Only cations with an appropriate radius fit the cavity perfectly, larger ones have to deform it, while smaller ones find a nonoptimal coordination geometry [5–10].

Ionophores are most effective against Gram-positive bacteria. The cell of these bacteria is surrounded by the peptidoglycan layer which is porous and allows small molecules to pass through, reaching the cytoplasmic membrane, where the lipophilic ionophore rapidly dissolves into the membrane. Conversely, Gram-negative bacteria are separated from the environment and antimicrobial agents by a lipopolsaccharide layer, outer membrane, and periplasmic space [2]. *Escherichia coli*, Gram-negative bacteria of significant importance to food safety and human health, is insensitive to ionophore addition, unless the outer membrane is removed [11].

In this paper, we present structure and properties of several naturally occurring polyether antibiotics.

The molecular structures of polyether ionophores presented below are visualized using X-ray data deposited in Cambridge Structural Database.

## 2. Structures and Biological Properties of Several Ionophores

### 2.1. Alborixin

**Figure 1:**
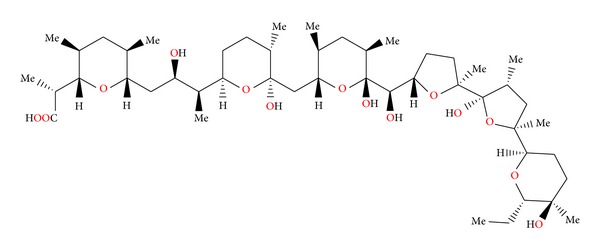
Structure of alborixine.

**Figure 2:**
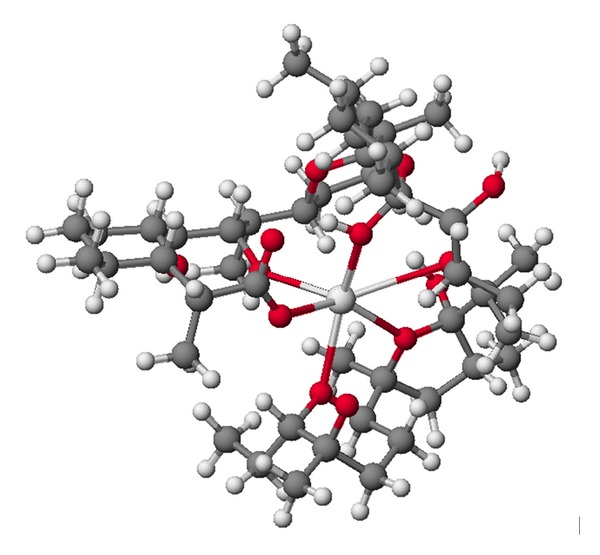
Structure of 6-demethyl-alborixin complex with sodium cation.

 Alborixin (Figure 1) was first isolated from fermenting a fermentation of *Streptomyces hygroscopicus* [12]. Its structure was determined by X-ray analysis of its potassium salt [13]. A crystal structure of 6-demethyl-alborixin complex with sodium cation (Figure 2) has also been reported [14]. Alborixin is active against Gram-positive bacteria. The value of LD_50_ of alborixin determined in mice subcutaneously is 150 mg/kg [15].

### 2.2. Antibiotic 6016

**Figure 3:**
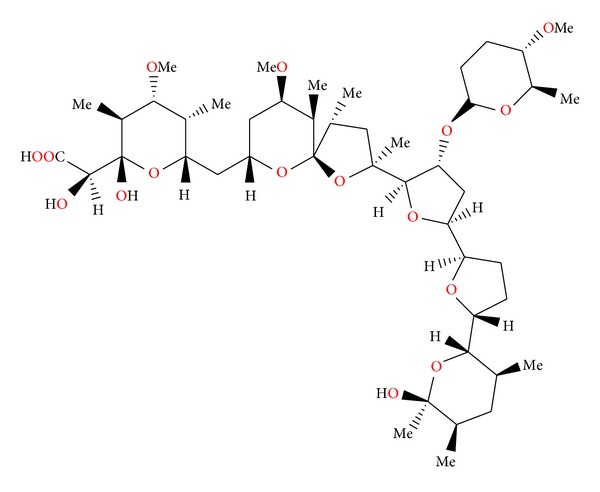
Structure of antibiotic 6016.

**Figure 4:**
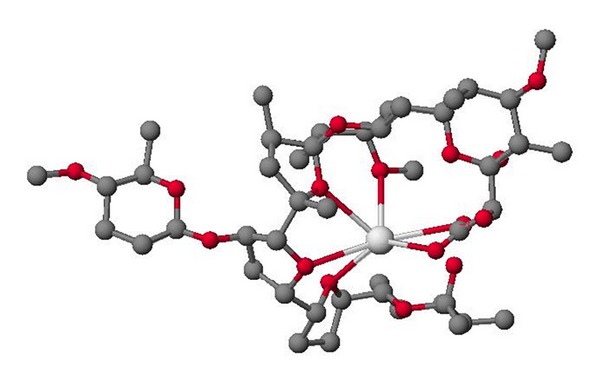
Crystal structure of antibiotic 6016 thallium salt.

Antibiotic 6016 (Figure 3) was first isolated from the culture of *Streptomyces albus* strain No. 6016 [16]. Molecular structure (Figure 4) was determined by X-ray analysis of its thalium salt [17]. Antibiotic 6016 is active against Gram-positive bacteria including mycobacteria. No activity was observed against Gram-negative bacteria and fungi. The toxicity of antibiotic 6016 in mice was also examined. The LD_50_ is 23 mg/kg intraperitoneally and 63 mg/kg orally. The anticoccidial evaluation of antibiotic 6016 was carried out on chickens infected with *Eimeria tenella* oocyst. It was effective in reducing mortality of chickens and increasing average body weight of treated infected chickens compared to untreated infected controls [16].

### 2.3. Calcimycin, Cezomycin, and X-14885A

**Figure 5:**
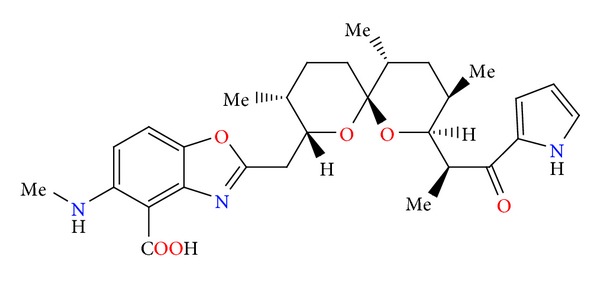
Structure of calcimycin.

**Figure 6:**
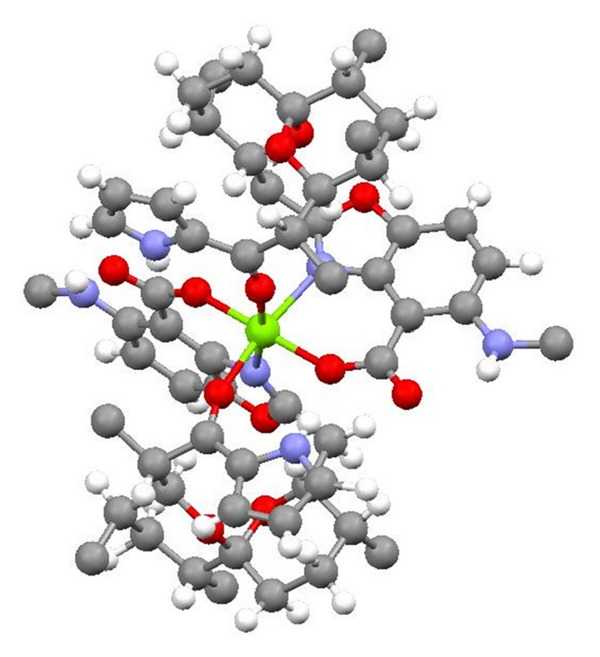
Crystal structure of 2 : 1 complex of calcimycin with the magnesium cation.

Calcimycin (Figure 5) was first isolated from a fermentation of *Streptomyces chartreuses *[18]. This antibiotic is able to bind divalent cations with a preference for the complexation of calcium cation [19]. Complexes of calcimycin with Mg^2+^, Ni^2+^, and Zn^2+^ cations on 2 : 1 stoichiometry are also described [20–22]. Structure of complex with Mg^2+^ is presented in Figure 6.

By equilibrating calcium ion across the inner membrane of mitochondria, calcimycin can uncouple oxidative phosphorylation. Various biological processes which require the presence of a calcium ion, such as thyroid secretion and insulin release, have been stimulated using this ionophore [23, 24]. Because of the fluorescent properties of calcimycin, it has been used also as a probe for divalent cations in artificial and biological membranes and to determine the mode of action of ionophore-mediated divalent cation transport [25].

Calcimycin is an efficient antibiotic against Gram-positive bacteria and inactive towards Gram-negative species. This difference in activity is attributed to the outer membrane of Gram-negative bacteria which is presumably impermeable to these highly hydrophobic compounds [26].

**Figure 7:**
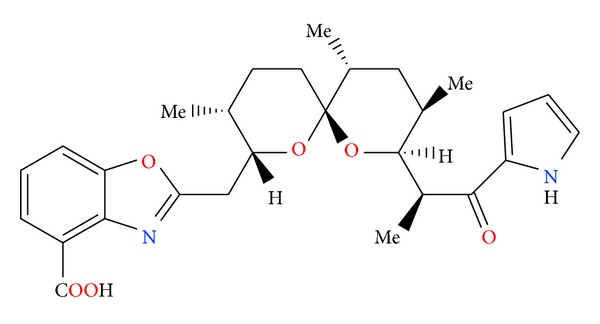
Structure of cezomycin.

**Figure 8:**
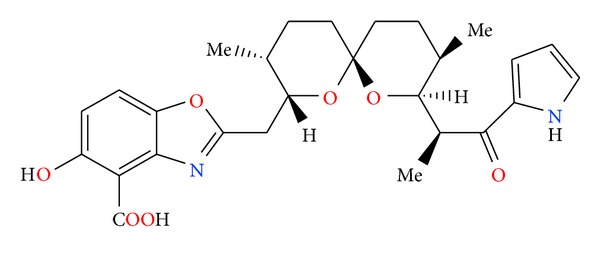
Structure of X-14885A.

**Figure 9:**
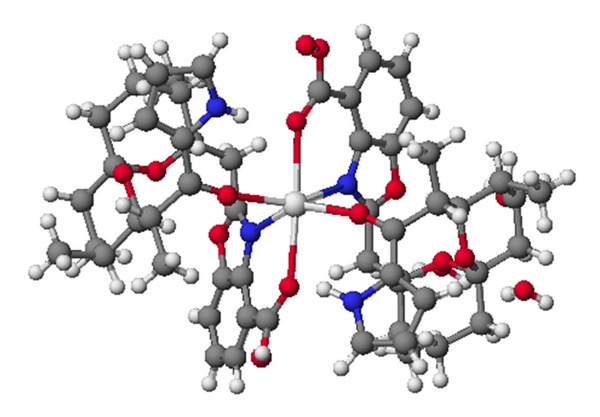
Crystal structure of 2 : 1 complex of 11-demethyl-cezomycin complex with the sodium cation.

There are two compounds structurally related to calcimycin: cezomycin (Figure 7) also known as demethyloamino-calcimycin and X-14885A (Figure 8), which has one methyl group less on the spiroketal and a hydroxyl group instead of a methyloamino group present in calcimycin. Both compounds are isolated from the same strain as calcimycin. The crystal structure of cezomycin (Figure 9) was determined as its 11-demethyl complex with the sodium cation of the stoichiometry 2 : 1 [27].

 Antibiotic X-14885A exhibits *in vitro* activity at concentrations less than 1 *μ*g/mL against such Gram-positive bacteria as *Staphylococcus aureus* and *Bacillus subtilis* and the spirochete responsible for swine dysentery *Treponema hyodysenteriae *[28].

### 2.4. Cationomycin

**Figure 10:**
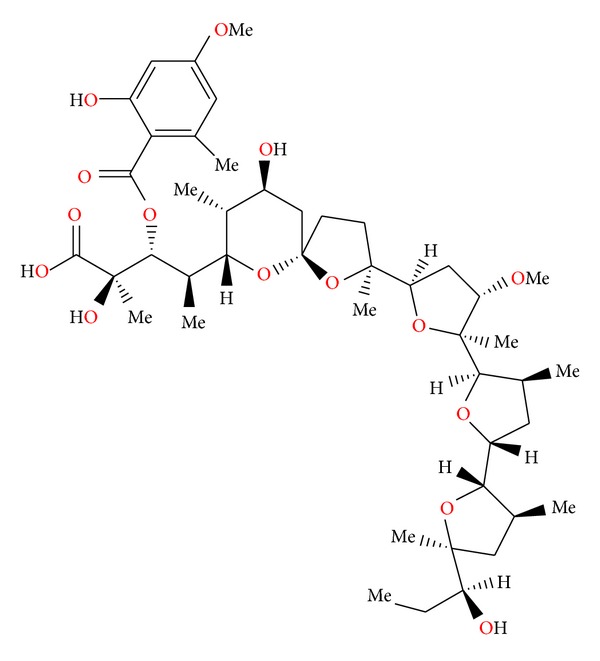
Structure of cationomycin.

**Figure 11:**
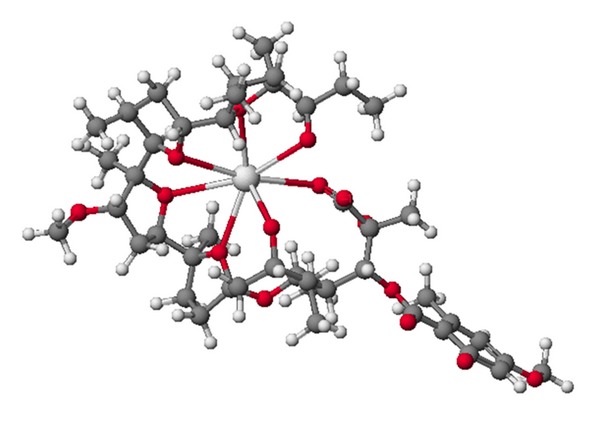
Crystal structure of cationomycin thallium salt.

Cationomycin (Figure 10) is an ionophore isolated from *Actinomadura azurea*. Crystal structure of cationomycin (Figure 11) was determined by X-ray analysis of its thallium salt [29]. Cationomycin contains an unusual aromatic side fragment, which is important for biological activity. When this chain is removed, the activity of cationomycin is reduced. A large group of derivatives was prepared after deacylation, but only anisyl analogue was more active than cationomycin [30, 31].

Kinetic studies showed that cationomycin transported the potassium cation more rapidly than the sodium cation, and the more stable complex was formed with potassium at the water/membrane interface [32].

### 2.5. Endusamycin

**Figure 12:**
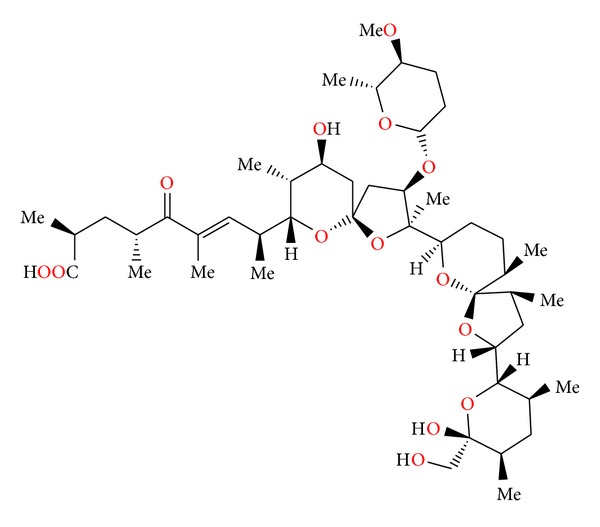
Structure of endusamycin.

**Figure 13:**
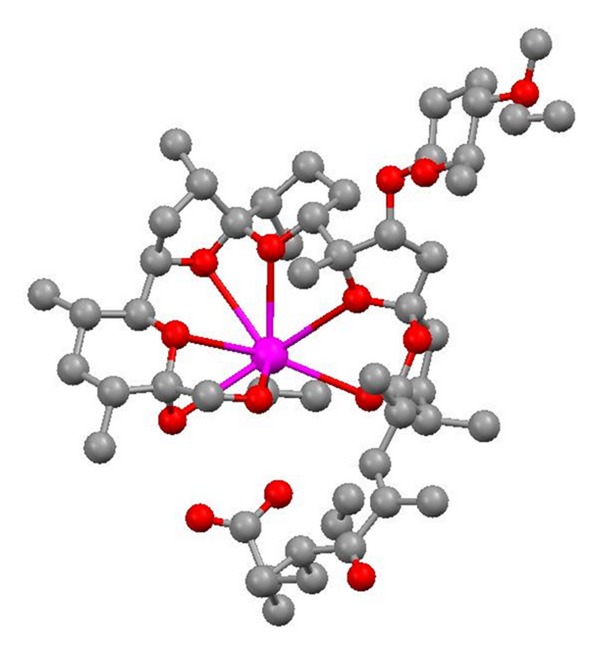
Crystal structure of endusamycin rubidium salt.

Endusamycin (Figure 12) was isolated from *Streptomyces endus*. The crystal structure (Figure 13) was determined by X-ray analysis of its rubidium salt [33].

Endusamycin has a good spectrum of activity against Gram-positive bacteria and good activity against many anaerobes and organisms such as *Treponema hyodysenteriae*. Endusamycin was active against *Eimeria tenella* and *Eimeria acervulina* coccidia when administrated in feed from 10 to 40 *μ*g/g. Chickens were protected from lesions but showed poor weight gains and feed intake. Endusamycin has LD_50_ of 7.5 mg/kg orally in male rats [33].

Endusamycin also induced a change in the proportion of volatile fatty acids (acetate, propionate, and butyrate) produced in the rumen by increasing the molar proportion of propionate in the rumen fluids [33].

### 2.6. Mutalomycin

**Figure 14:**
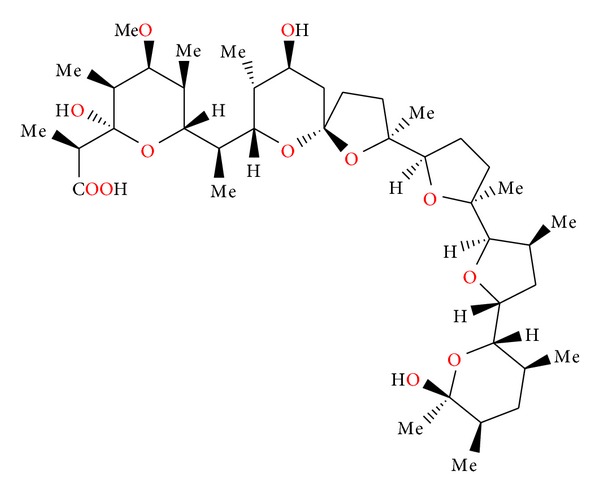
Structure of mutalomycin.

**Figure 15:**
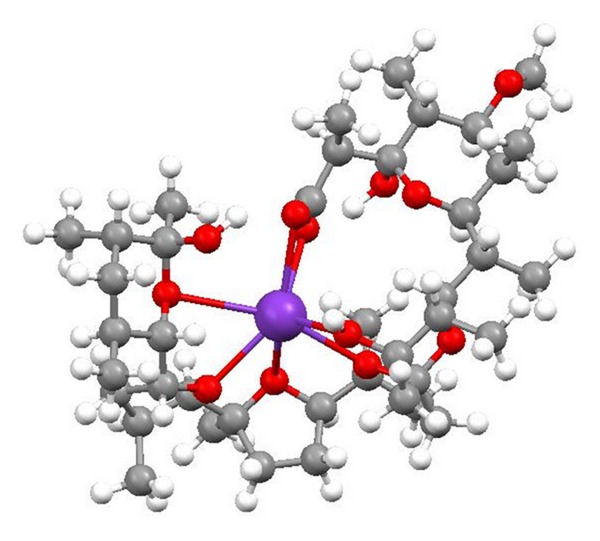
Crystal structure of 28-epimutalomycin potassium salt.

Mutalomycin (Figure 14) was first isolated from a strain of *Streptomyces mutabilis* [34]. Crystal structure of its epimer, 28-epimutalomycin potassium salt (Figure 15), was reported [35].

 Mutalomycin possesses antibiotic activity against Gram-positive bacteria and also exhibits an anticoccidial activity in chickens similar to other polyether antibiotics. It is effective in reducing mortality and in increasing the average body weight of chickens infected with *Eimeria tenella* and other *Eimeria* species [34].

### 2.7. Ionomycin

**Figure 16:**
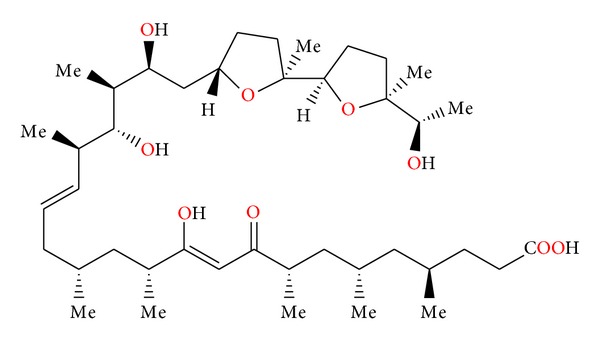
Structure of ionomycin.

**Figure 17:**
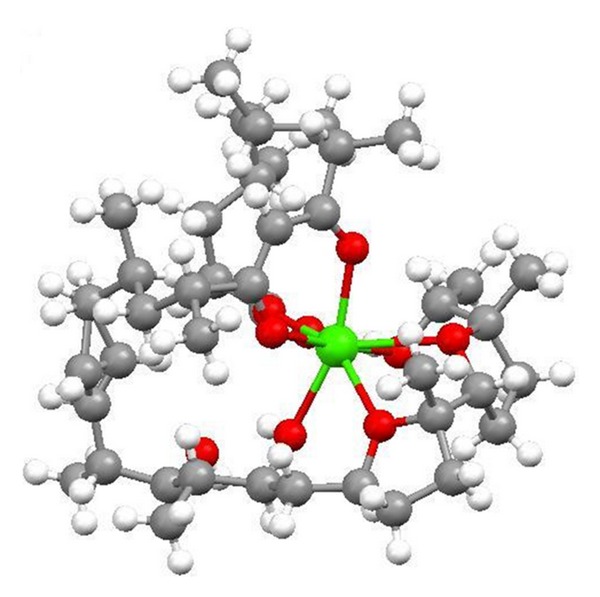
Crystal structure of ionomycin complex with the calcium cation.

Ionomycin (Figure 16) was first isolated from *Streptomyces conglobatus* [36]. Crystal structure of its complex with the calcium cation (Figure 17) was reported [37].

 Ionomycin is capable of extracting calcium ion from the aqueous phase into an organic phase. This antibiotic also acts as a mobile ion carrier transporting the cation across a solvent barrier. The divalent cation selectivity order for ionomycin as determined by ion competition experiments was found to be Ca^2+^ > Mg^2+^≫ Sr^2+^ = Ba^2+^, where the binding of strontium and barium by the antibiotic is insignificant [36].

 Ionomycin, like other polyether antibiotics, is active primarily against Gram-positive bacteria with no demonstrable activity against Gram-negative bacteria. The acute toxicity of ionomycin, LD_50_, administered subcutaneously to mice is 28 mg/kg [38].

### 2.8. K-41

**Figure 18:**
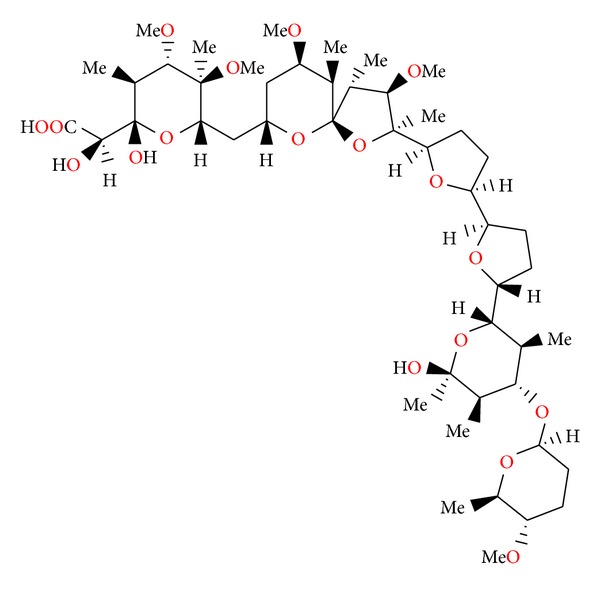
Structure of K-41.

 K-41 (Figure 18) was first isolated from *Streptomyces hygroscopicus* [39]. The molecular structure of this antibiotic was established by X-ray analysis of the sodium salt of its *p*-bromobenzoate [40].

 K-41 is active against Gram-positive bacteria [40]. K-41 showed also antimalarial activity against the drug-resistant strain of *Plasmodium falciparum* and was more potent than clinically used antimalarial drugs: artemisinin, chloroquine, and pyrimethamine [41].

### 2.9. Kijimicin

**Figure 19:**
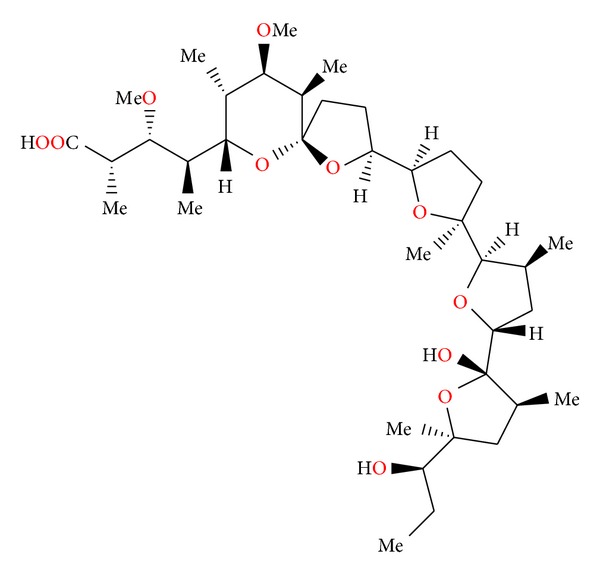
Structure of kijimicin.

**Figure 20:**
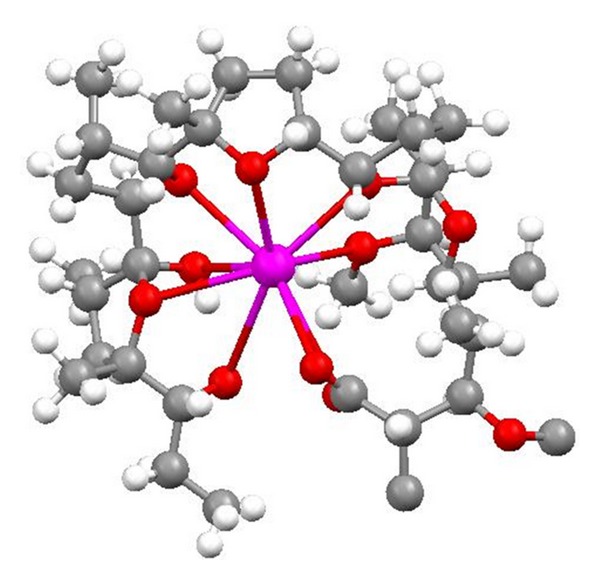
Crystal structure of kijimicin rubidium salt.

Kijimicin (Figure 19) was found in the culture filtrate of an *Actinomadura *sp. MI215-NF3. Its crystal structure (Figure 20) was established by X-ray analysis of its rubidium salt [42].

 Kijimicin inhibits mainly the growth of Gram-positive bacteria and shows activity against *Eimeria tenella*. The acute toxicity of the antibiotic in mice is 56 mg/kg [43]. Kijimicin was also examined for its HIV inhibitory activity, was proved to inhibit HIV replication in both T-cell and monocyte lineage cell lines, and was shown to be active in *in vitro* assays against both acute and chronic infections [43].

### 2.10. Lasalocid A and Its Analogues

**Figure 21:**
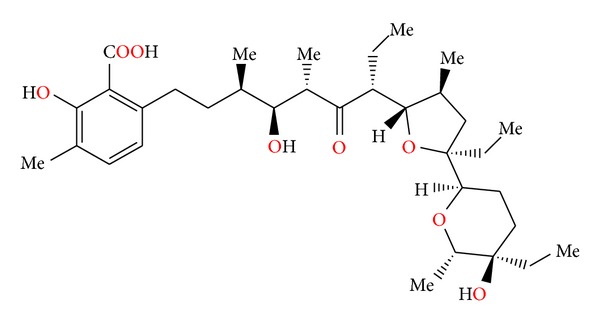
Structure of lasalocid.

**Figure 22:**
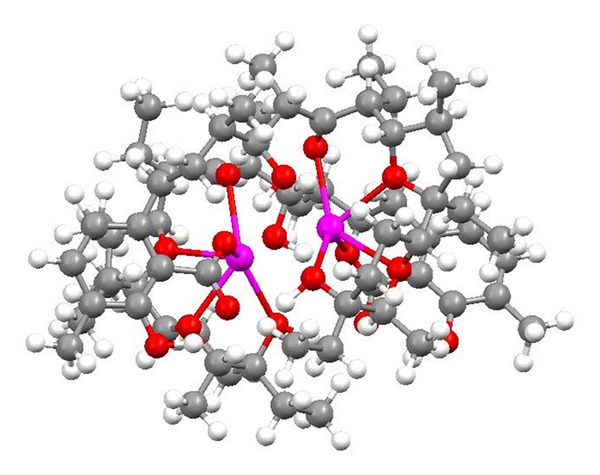
Crystal structure of lasalocid silver salt on 2 : 2 stoichiometry.

**Figure 23:**
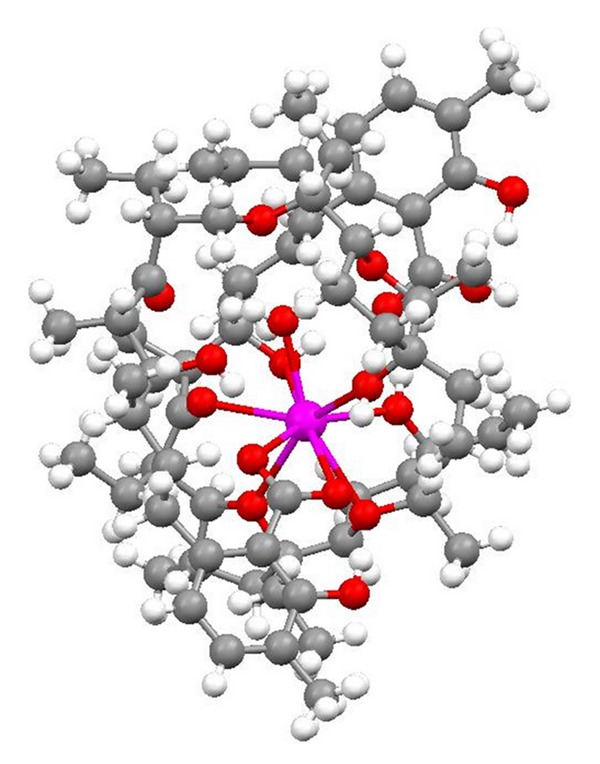
Crystal structure of 2 : 1 complex of lasalocid with the strontium cation.

**Figure 24:**
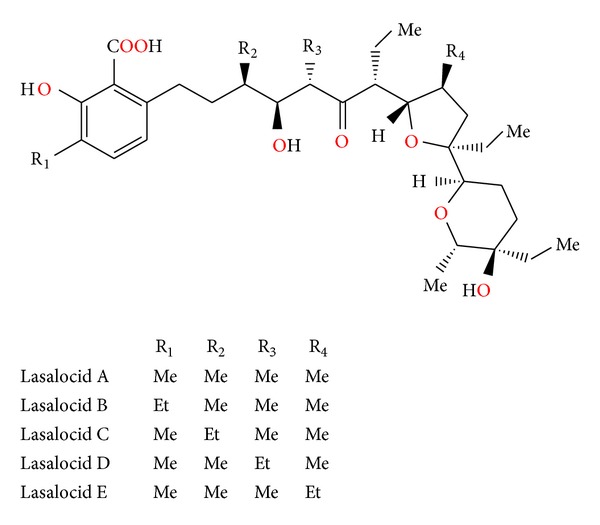
Lasalocid acid analogues.

Lasalocid (Figure 21) was first isolated from *Streptomyces lasaliensis* [44]. Crystal structures of lasalocid acid barium, silver (Figure 22), and strontium (Figure 23) salts were determined [45, 46]. Monomeric unit of Lasalocid thallium salt is stabilized by strong intramolecular aryl-Tl type-metal half sandwich bonding interactions [47]. Homologs of lasalocid (Figure 24) acid were also described [48].

**Figure 25:**
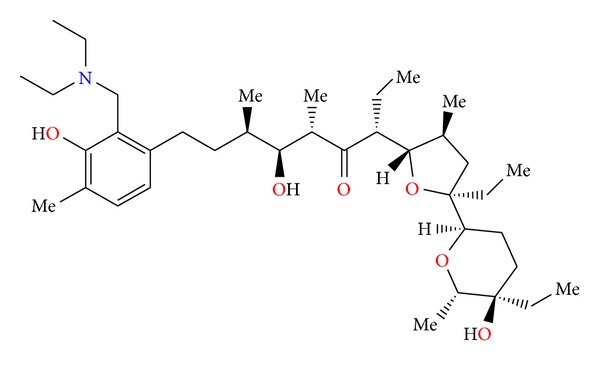
Structure of lasalocid acid Mannich base.

**Figure 26:**
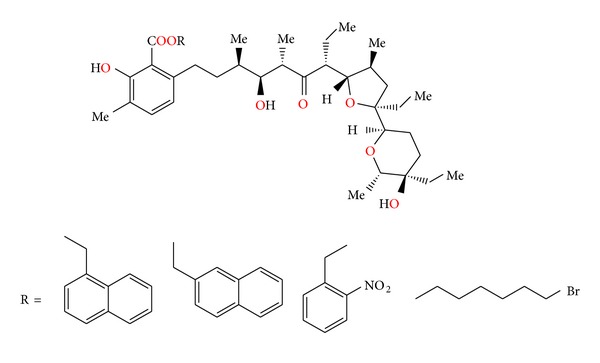
Structures of lasalocid acid esters.

**Figure 27:**
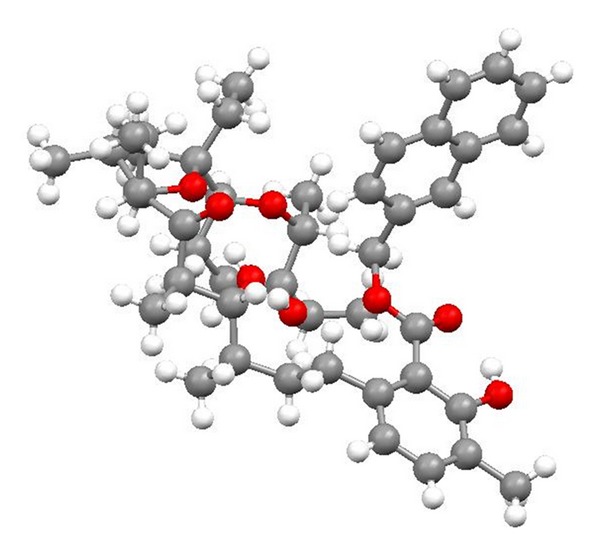
Structure of lasalocid 2-naphthylmethyl ester.

**Figure 28:**
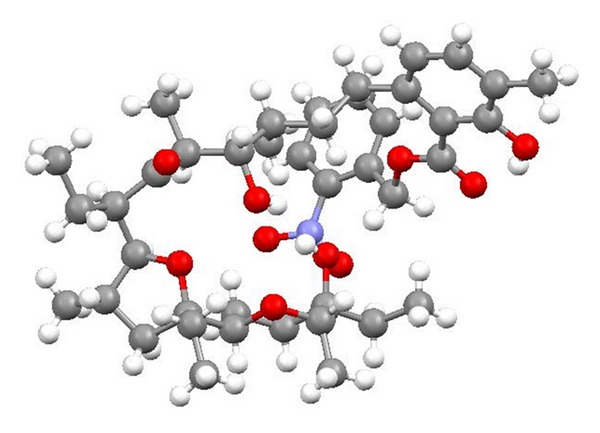
Crystal structure of lasalocid orthonitrobenzyl ester.

Chemistry of lasalocid was extensively investigated. Treatment of the free acid with diethylamine and paraformaldehyde in toluene, employing Dean—Stark conditions, gave the Mannich base (Figure 25) [49]. Four ester derivatives of lasalocid were obtained (Figure 26), and their crystal structures (Figures 27 and 28) were also reported [50–53].

**Figure 29:**
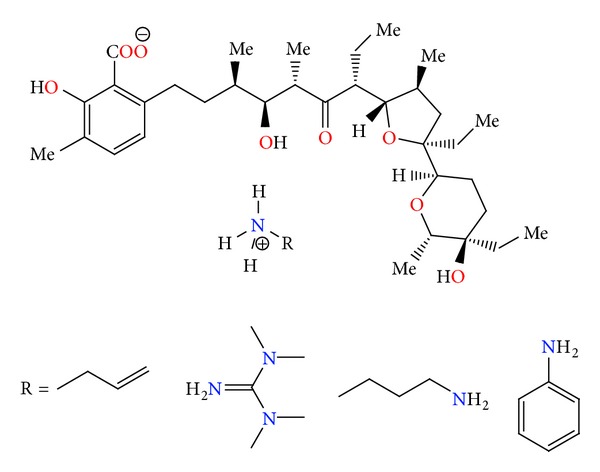
Lasalocid acid complexes with several amines: allylamine, 1,1,3,3-tetramethylguanidine, *N*-butylamine, and phenylamine.

**Figure 30:**
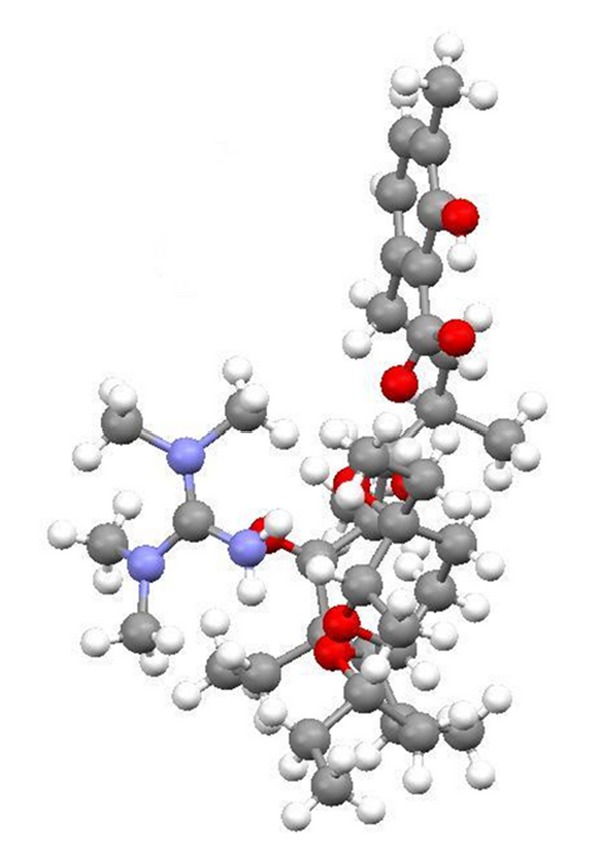
Crystal structure of lasalocid complex with tetramethylguanidine.

**Figure 31:**
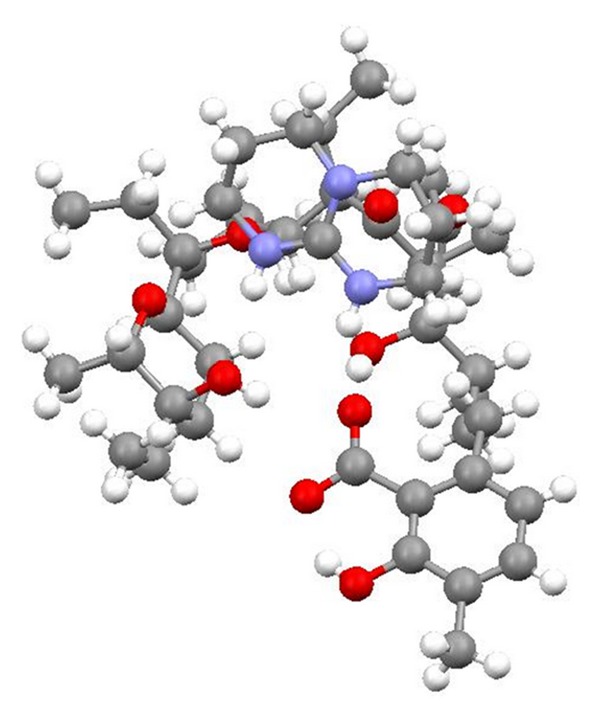
Crystal structure of lasalocid complex with TBD.

 Lasalocid is able to form complexes with amines and its several complexes have been obtained (e.g., with allylamine, 1,1,3,3-tetramethylguanidine, TBD, phenylamine, and *N*-butylamine) (Figure 29), and their crystal structures (Figures 30 and 31) were reported. Lasalocid is active against Gram-positive bacteria. Complex of lasalocid acid with allylamine is even more active than pure lasalocid acid. Lasalocid sodium salt (Bovatec, Avate) is used in a veterinary to prevent coccidiosis in poultry and to improve nutrient absorption and feed efficiency in ruminants [54–57].

 Recently, two complexes of lasalocid (with phenylamine and butylamine) were tested *in vitro *for cytotoxic activity against human cancer cell lines: A-549 (lung), MCF-7 (breast), HT-29 (colon), and mouse cancer cell line P-388 (leukemia). It was found that lasalocid and its complexes are strong cytotoxic agents towards cell lines. The cytostatic activity of the compounds studied is greater than that of cisplatin, indicating that lasalocid and its complexes are promising candidates for new anticancer drugs [57].

### 2.11. Semduramicin and CP-120509

**Figure 32:**
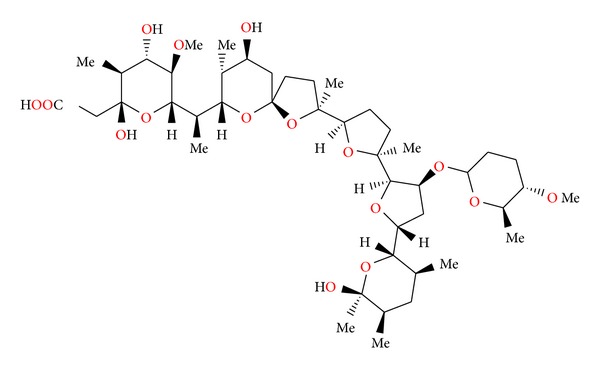
Structure of semduramicin.

Semduramicin (Figure 32) was isolated from *Actinomadura roserufa* [58]. The anticoccidial activity tests of semduramicin against laboratory isolates of five species of poultry Eimeria have shown that this antibiotic is active from 20 to 30 ppm concentrations. Semduramicin was well tolerated when coadministered with tiamulin [59].

**Figure 33:**
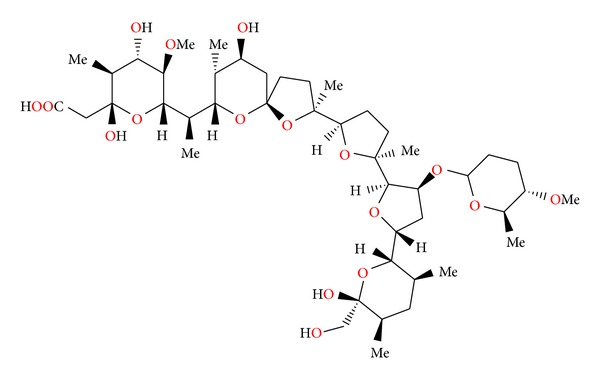
Structure of CP-120509.

 Antibiotic CP-120509 (Figure 33) is also isolated from *Actinomadura roserufa* and is structurally related to semduramicin. Its crystal structure was reported [60]. CP-120509 exhibited *in vitro* activity against certain Gram-positive bacteria and the spirochete *Serpulina hyodysenteriae*, the causative agent of swine dysentery, but was inactive against Gram-negative bacteria. It afforded excellent anticoccidial activity against *Eimeria acervulina* in chickens at levels between 30 and 60 mg/kg in fed [60].

### 2.12. Tetronasin

**Figure 34:**
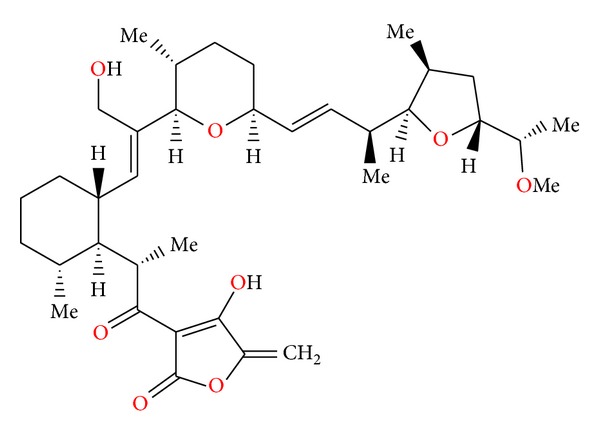
Structure of tetronasin.

Tetronasin (Figure 34) was isolated from *Streptomyces longisporoflavus. *The structure of this antibiotic has been determined by X-ray analysis of the 4-bromo-3,5-dinitrobenzoyl derivative [61].

 Gram-positive bacteria are sensitive to the tetronasin and were unable to adapt to grow in the presence of this antibiotic. Gram-negative bacteria were more resistant. An *in vivo* trial with cattle and *in vitro* growth experiments indicated that the effect of tetronasin on ciliate protozoa was minor. *In vitro* experiments measuring hydrogen production by *Neocallimastix frontalis *suggested that this fungus would be unable to survive in ruminants receiving tetronasin [62].

### 2.13. Zincophorin and CP-78545

**Figure 35:**
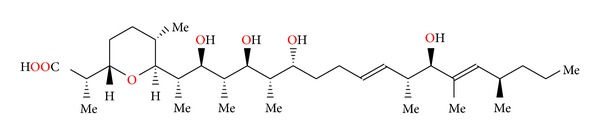
Structure of zincophorin.

**Figure 36:**
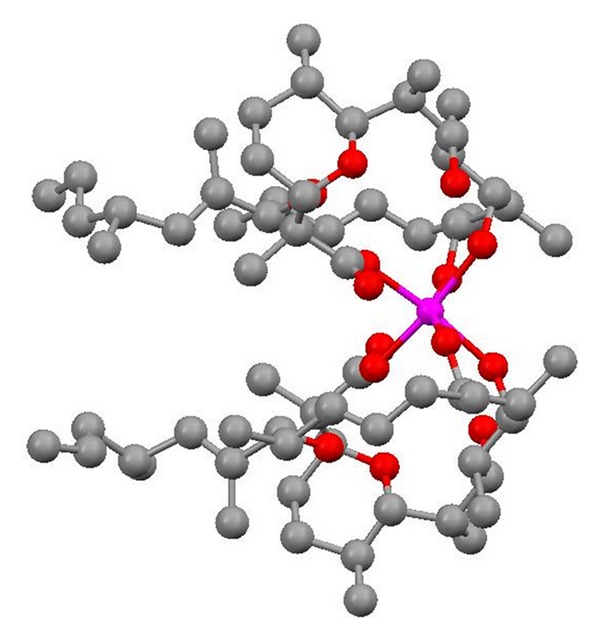
Crystal structure of zincophorin magnesium salt.

Zincophorin (Figure 35) was first isolated from *Streptomyces griseus*. Its crystal structure was determined by X-ray analysis of its magnesium salt (Figure 36) [63].

 Zincophorin is able to complex divalent cations, with the stability order of zinc *≈* cadmium > magnesium > strontium *≈* barium *≈* calcium. 

 Zincophorin showed good *in vitro* activity against Gram-positive bacteria. It also inhibited methane production and favourably altered volatile fatty acid ratios in *in vitro *rumen fermentations. It showed some anticoccidial activity against *Eimeria tenella* in chicks [63].

**Figure 37:**
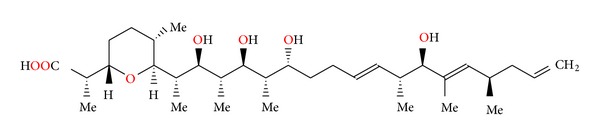
Structure of CP-78545.

**Figure 38:**
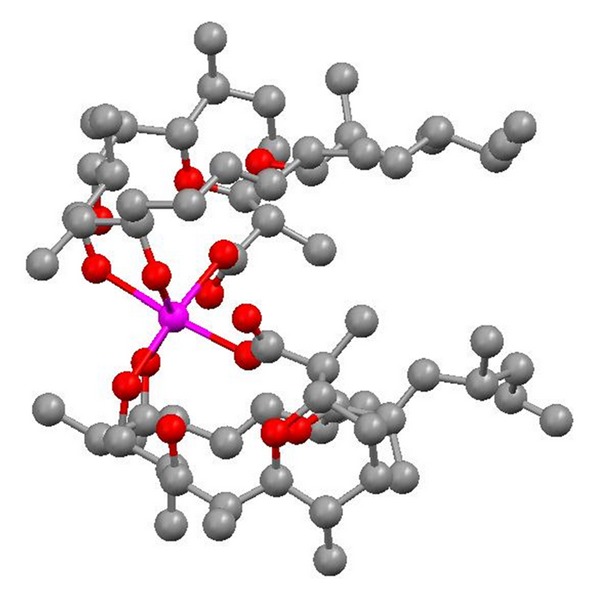
Crystal structure of CP-78545 cadmium salt.

 Antibiotic CP-78545 (Figure 37) also isolated from *Streptomyces griseus *is structurally related to zincophorin. Additional unsaturated bond is present in CP-78545 compared to zincophorin. Crystal structure of CP-78545 was determined by X-ray analysis of its cadmium salt (Figure 38).

 CP-78545 exhibited *in vitro* antibiotic activity against certain Gram-positive bacteria such as *Bacillus*, *Staphylococcus*, and *Streptococcus* as well as the anaerobe *Treponema hyodysenteriae* (the causative agent of swine dysentery), but no activity towards Gram-negative bacteria. It was active *in vitro* against a coccidium *Eimeria tenella* in a tissue culture assay, but it was inactive *in vivo* at levels between 100 and 200 mg/kg in feed versus *Eimeria tenella* coccidial infections in chickens [64].

### 2.14. Salinomycin SY-1, SY-2, SY-4, and SY-9

**Figure 39:**
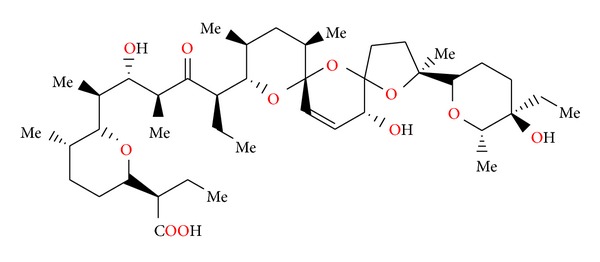
Structure of salinomycin.

**Figure 40:**
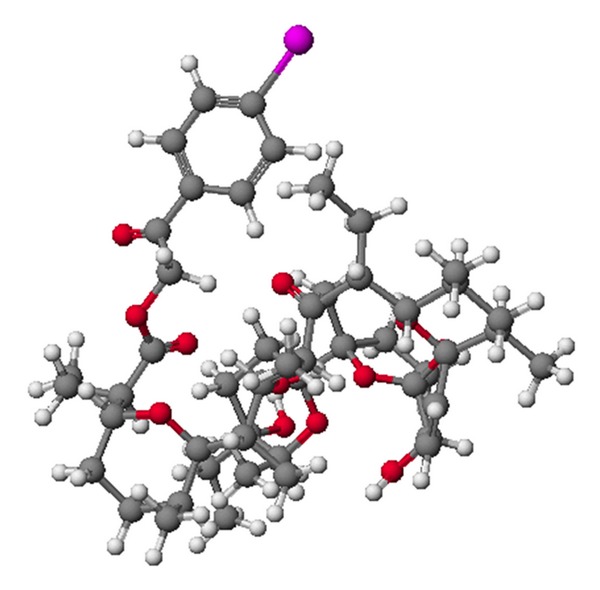
Crystal structure of salinomycin p-iodophenacyl ester.

**Figure 41:**
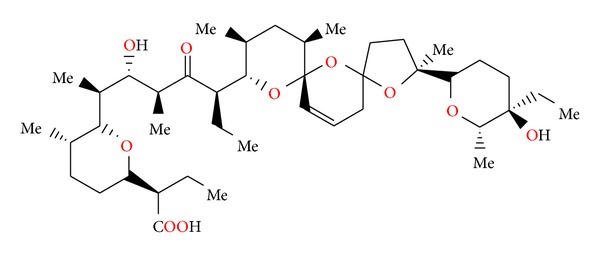
Structure of SY-1.

**Figure 42:**
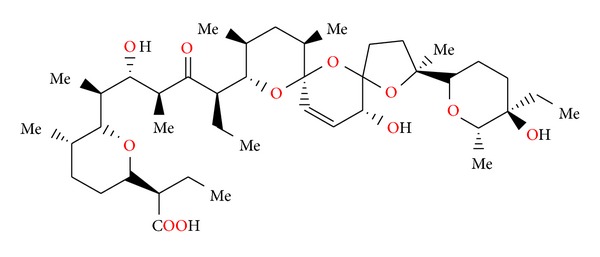
Structure of SY-2.

**Figure 43:**
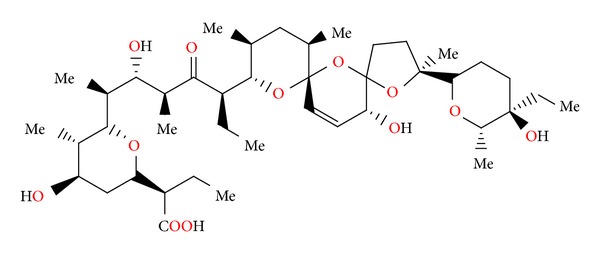
Structure of SY-4.

**Figure 44:**
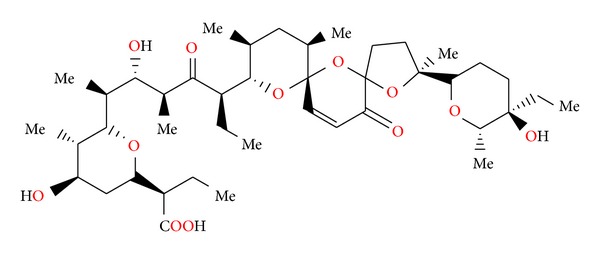
Structure of SY-9.

**Figure 45:**
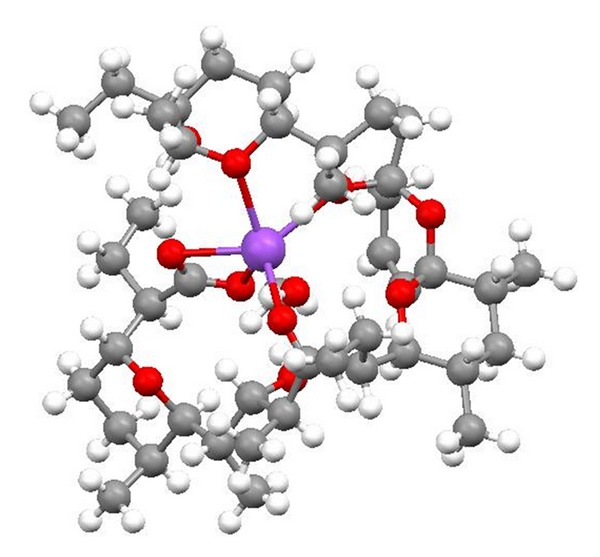
Crystal structure of SY-1 with the sodium cation.

**Figure 46:**
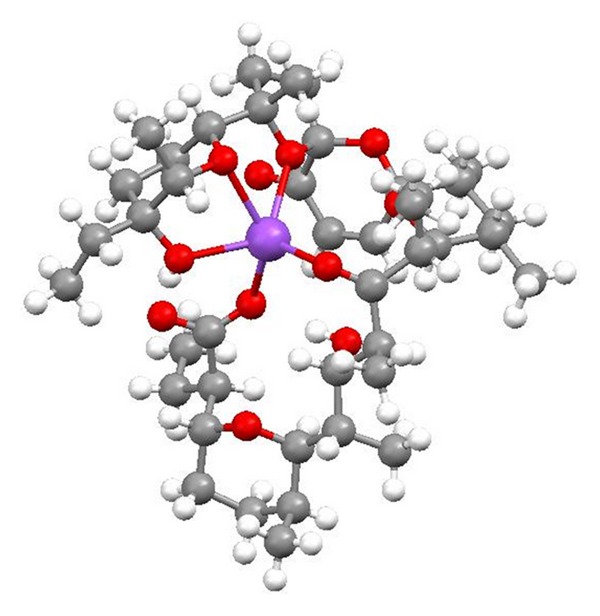
Crystal structure of SY-9 with the sodium cation.

**Figure 47:**
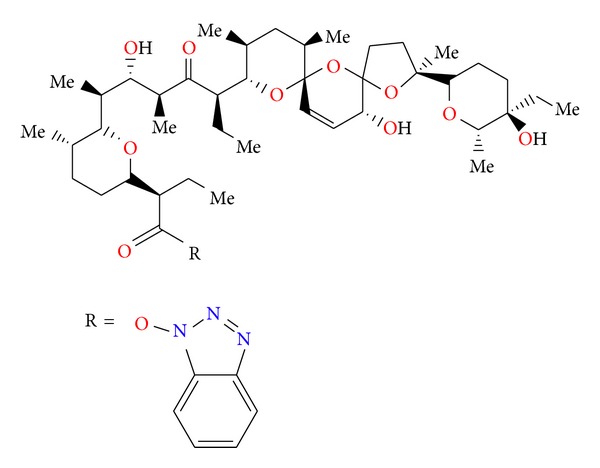
Structure of benzotriazole ester of salinomycin.

**Figure 48:**
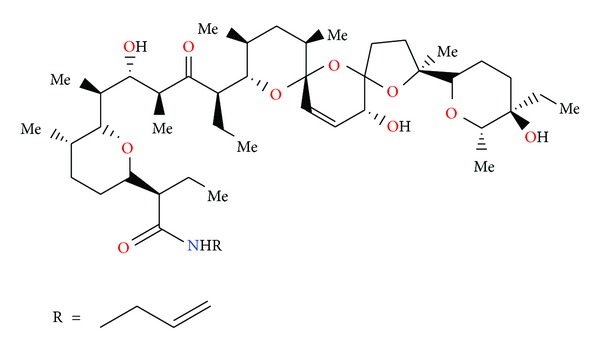
Structure of allyl amide of salinomycin.

**Figure 49:**
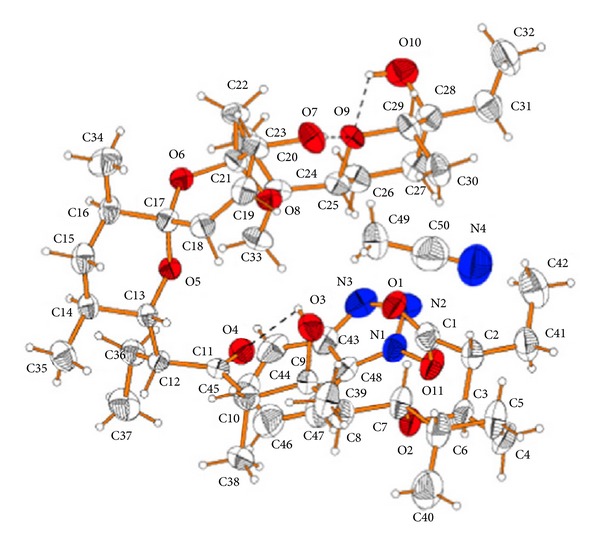
Crystal structure of salinomycin benzotriazole ester acetonitrile solvate.

**Figure 50:**
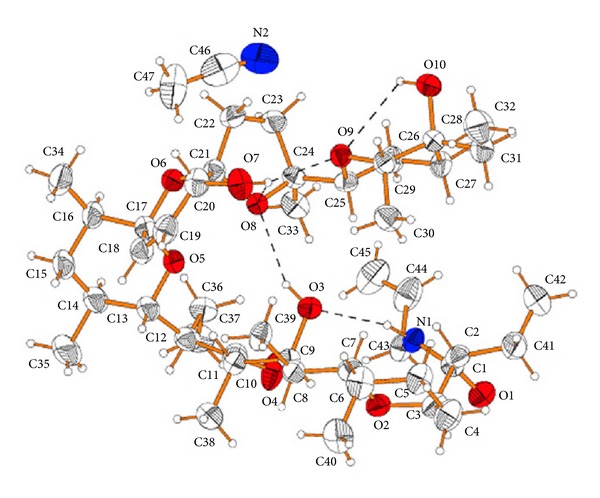
Crystal structure of salinomycin allyl amide acetonitrile solvate.

Salinomycin (Figure 39) was isolated from *Streptomyces albus*. Its crystal structure (Figure 40) has been established by X-ray analysis of its p-iodophenacyl ester [65]. Compounds structurally related to salinomycin have also been obtained and described. SY-1 (Figure 41) is 20-deoxysalinomycin. SY-2 (Figure 42) is C-17 epimer of SY-1 [66]. SY-4 (Figure 43) is 5-hydroxysalinomycin [67], and SY-9 (Figure 44) is 20-oxosalinomycin. Crystal structures of SY-1 (Figure 45) and SY-9 (Figure 46) were reported [68, 69]. Two other ester and amide derivatives of salinomycin (Figures 47 and 48) were obtained, and their crystal structures (Figures 49 and 50) were reported [70, 71].

The relative affinity of salinomycin for complex formation with various cations decreases in the order K^+^ > Na^+^ > Cs^+^ > Sr^2+^> Ca^2+^, Mg^2+^. Salinomycin has been shown to transport monovalent cations more effectively than divalent cations from an aqueous buffer into an organic solvent [72].

 Salinomycin is active against Gram-positive bacteria including mycobacteria and some filamentous fungi. No activity was observed against Gram-negative bacteria and yeast. The acute toxicity of salinomycin in mice was examined, and its LD_50_ was 18 mg/kg intraperitoneally and 50 mg/kg orally. The anticoccidial estimation of salinomycin was carried out on chickens infected with *Eimeria tenella*. Salinomycin was effective in reducing mortality of chickens and in increasing the average body weight of treated infected chickens compared to those of untreated infected controls [73].

 Very recently, it has been shown that it is possible to selectively kill breast cancer stem cells using salinomycin. Its ability to kill cancer stem cells and apoptosis-resistant cancer cells may define salinomycin as a novel anticancer drug [74].

### 2.15. Monensin

**Figure 51:**
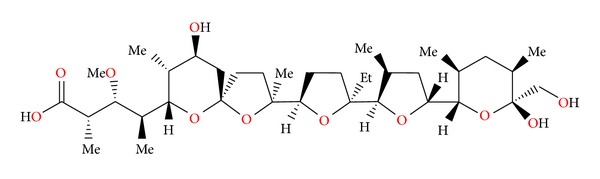
Structure of monensin.

**Figure 52:**
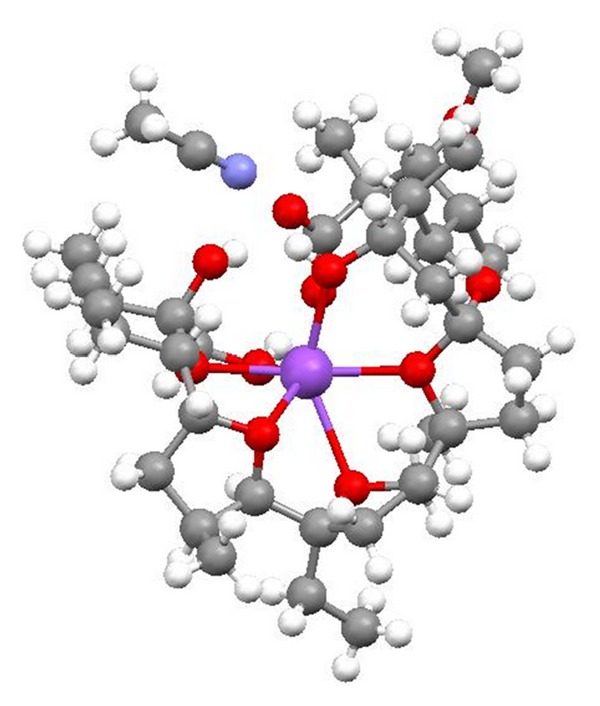
Crystal structure of monensin sodium salt acetonitrile solvate.

**Figure 53:**
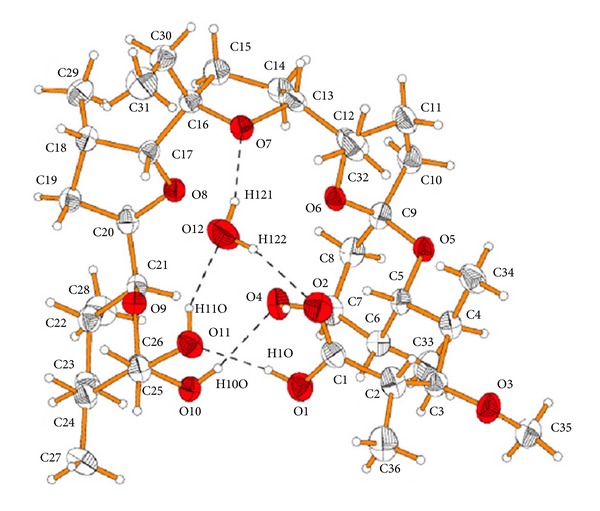
Structure of monensin hydrate.

**Figure 54:**
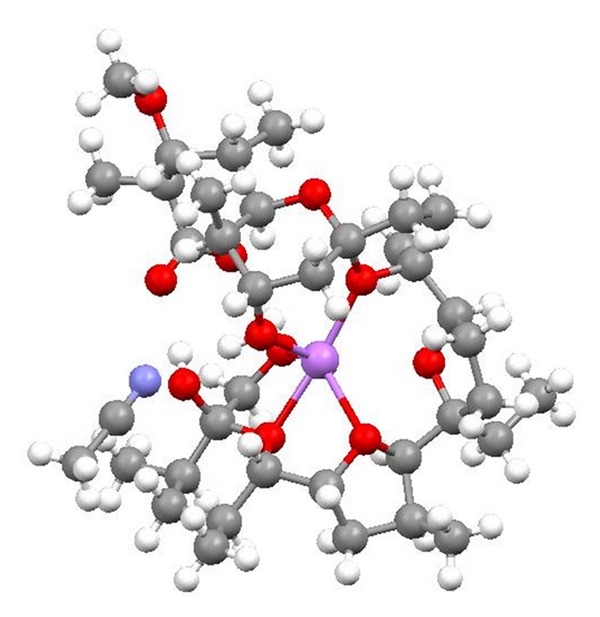
Crystal structure of monensin lithium salt acetonitrile solvate.

Monensin (Figure 51) was first isolated from *Streptomyces cinnamonensis* [75]. Crystal structures of monensin hydrate and its salts with sodium, lithium, and rubidium (Figures 52, 53, and 54) were reported [76–79]. In the last years Pantcheva et al. have shown that monensin (MON) can form two types of salt complex species with divalent metal cations.

**Figure 55:**
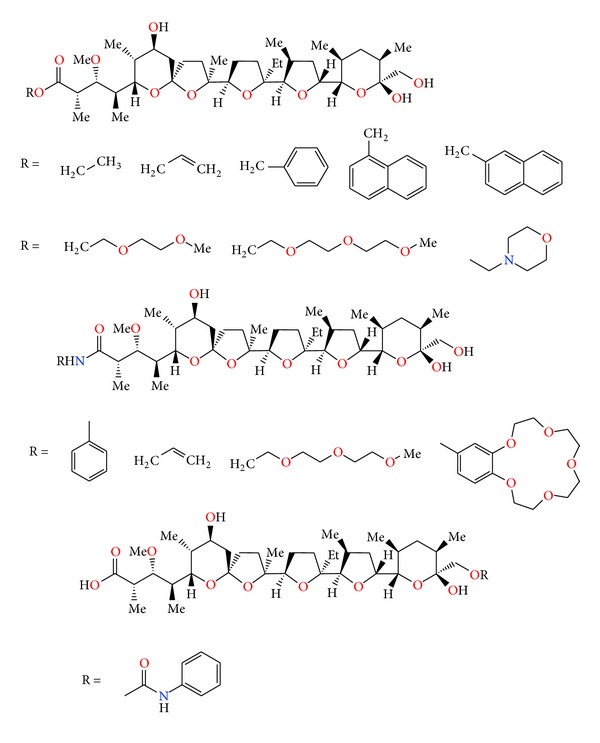
Structures of monensin ester amide and urethane derivatives.

**Figure 56:**
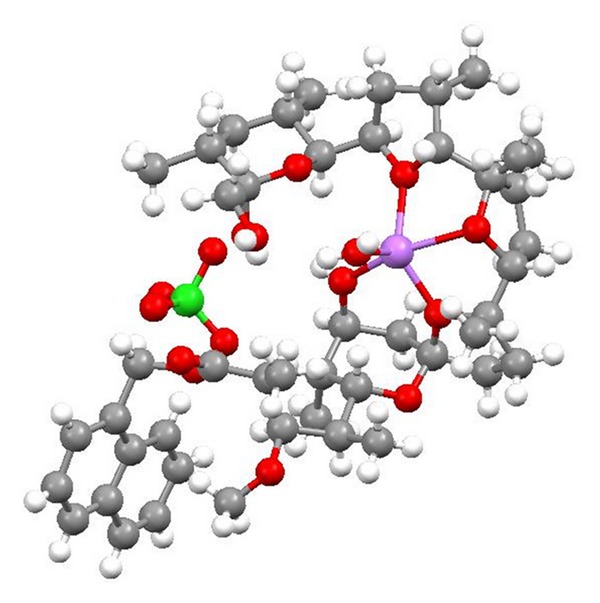
Crystal structure of monensin 1-naphtylmethyl ester with the lithium perchlorate.

**Figure 57:**
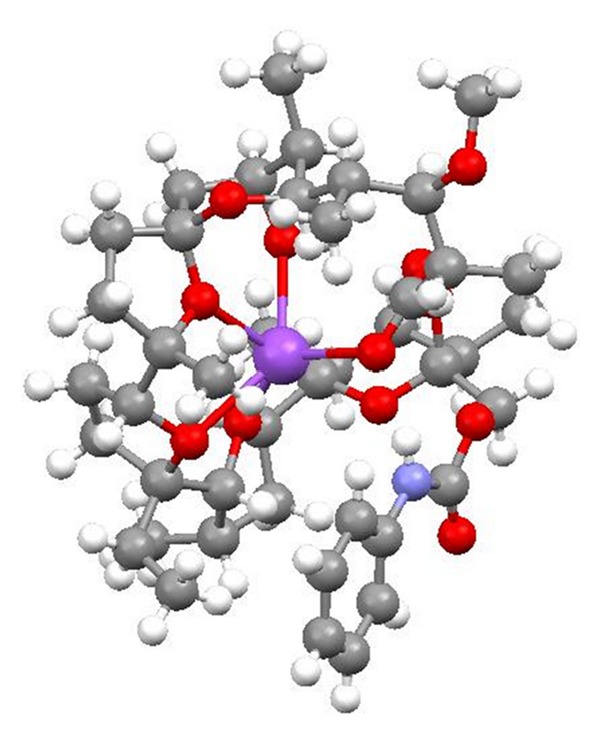
Crystal structure of monensin phenylurethane sodium salt.

In the first type, monensin sodium salt forms complexes with metal dichloride of [M(MON–Na)_2_]Cl_2_ *·*H_2_O stoichiometry, where M = Co^2+^, Mn^2+^ and Cu^2+^. In this type of structure, the divalent metal cation is tetrahedrally coordinated by oxygen atoms of two carboxylic groups of two monensin sodium salt molecules and by two chloride anions. The sodium cation remains in the hydrophilic cavity of the ligand and cannot be replaced by the transition metal cation. The second type of monensin complexes with the divalent metal cations is the neutral salt of the [M(MON)_2_ *·*(H_2_O)_2_] formula (M = Mg^2+^, Ca^2+^, Zn^2+^, Ni^2+^, Cd^2+^, and Hg^2+^). These salts consist of two monoanionic ligands (monensinates) bound in a bidentate coordination mode to the metal cation. These types of monensin salt complexes with divalent metal cation are untypical, because the etheric oxygen atoms do not play any role in the complexation of the cations. In contrast, in the typical complexes of monensin with monovalent metal cations, the etheric oxygen atoms of the monensin ligand are always involved in the complexation process [80–86]. A large group of monensin ester, amide, and urethane derivatives (Figure 55) was obtained and reported. Crystal structures of several of them (Figures 56 and 57) were also reported [87, 88]. Monensin exhibited *in vitro* antibiotic activity against certain Gram-positive bacteria such as *Bacillus*, *Staphylococcus*, and *Streptococcus*. No activity was observed against Gram-negative bacteria [89]. It was shown that monensin phenylurethane sodium salt shows a higher antibacterial activity against human pathogenic bacteria, including antibiotic-resistant *Staphylococcus aureus* and *Staphylococcus epidermidis* than the parent unmodified antibiotic monensin A [88]. Monensin blocks endocytosis and, therefore, impedes entry of toxic molecules. The drug also inhibits viral proliferation of RNA and DNA viruses such as vesicular stomatitis, influenza, and human polyomaviruses. Monensin also effectively abolishes viral DNA replication of mouse polyomavirus [90].

### 2.16. Ferensimycins A and B

**Figure 58:**
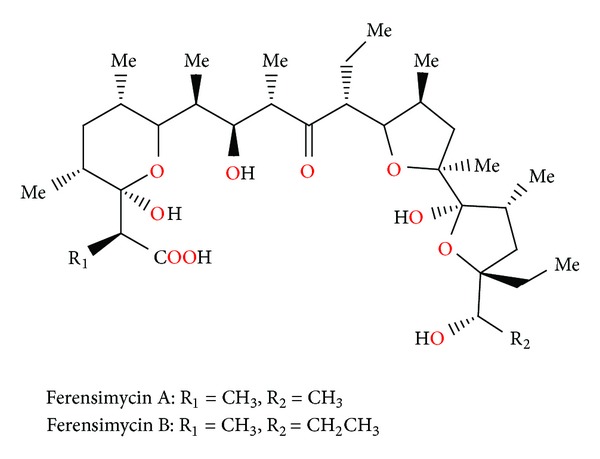
Structures of ferensimycins A and B.

Ferensimycins A and B (Figure 58) were isolated as their sodium salts from the fermentation broth of *Streptomyces* sp. No. 5057. Both antibiotics are active against Gram-positive bacteria but inactive against Gram-negative bacteria and fungi. The acute toxicity of these compounds in mice was examined, and the LD_50_ values of ferensimycin A and ferensimycin B were 30–50 mg/kg and 50 mg/kg, respectively [91].

### 2.17. CP-96797

**Figure 59:**
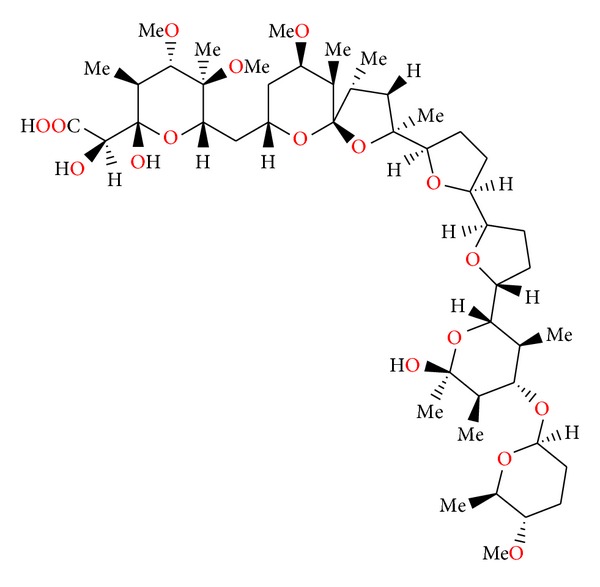
Structure of CP-96797.

 CP-96797 (Figure 59) was isolated from *Sterptomyces *sp. ATCC 55028. Crystal structure of the silver salt of this antibiotic was determined by X-ray analysis [92].

 CP-96797 sodium salt showed good activity against a number of Gram-positive bacteria, as well as the spirochete *Serpulina hyodysenteriae* (the causative agent of swine dysentery), but was inactive against Gram-negative bacteria. It afforded anticoccidial activity against *Eimeria tenella* in chickens at levels between 60 and 90 mg/kg in feed [92].

### 2.18. Octacyclomycin

**Figure 60:**
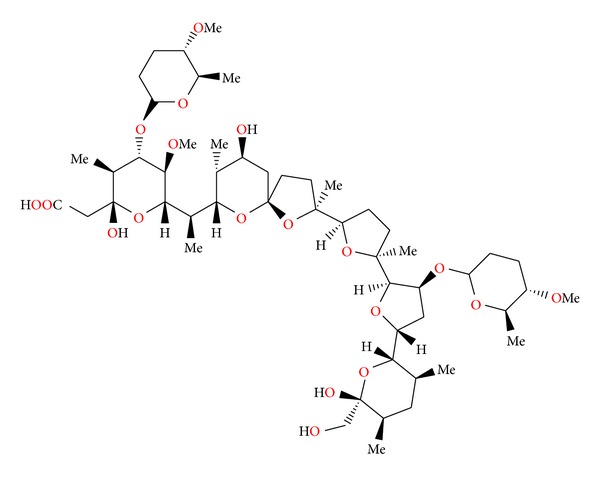
Structure of octacyclomycin.

Octacyclomycin (Figure 60) was isolated from *Streptomyces* sp. No. 82. This antibiotic showed cytocidal activity against B16 melanoma cells, and its IC_50_ value was 0.23 *μ*g/mL when the cells were exposed to the antibiotic for 3 days *in vitro*. On the other hand, octacyclomycin showed weak antimicrobial activity against Gram-positive bacteria such as *Staphylococcus aureus* and *Micrococcus luteus *at the concentration of 100 *μ*g/mL, whereas *Bacillus subtilis* was not affected at this concentration [93].

### 2.19. CP-91243 and CP-91244

**Figure 61:**
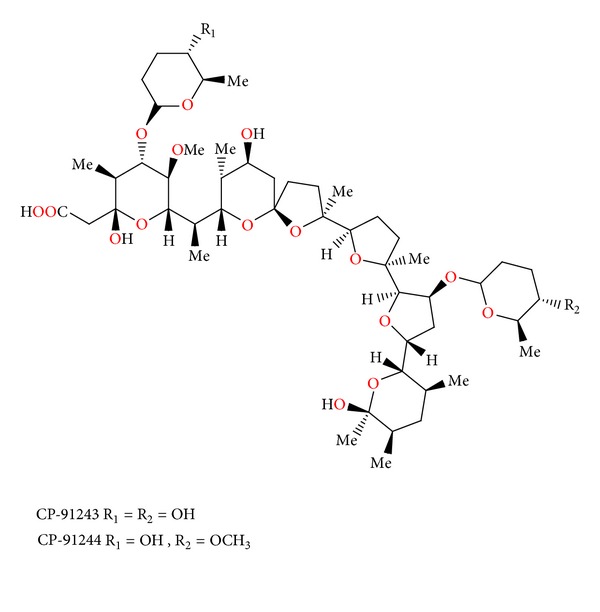
Structures of CP-91243 and CP-91244.

 CP-91243 and CP-91244 (Figure 61) were isolated from *Actinomadura roseorufa*. Both compounds exhibited *in vitro* activity against certain Gram-positive bacteria and the spirochete *Treponema hyodysenteriae* (the causative agent of swine dysentery), but were not active against Gram-negative bacteria. CP-91243 afforded anticoccidial activity against *Eimeria tenella* in chickens at 60 mg/kg in feed, and the less polar CP-91244 was about twice as active, 25 mg/kg in feed [94].

### 2.20. W341C

**Figure 62:**
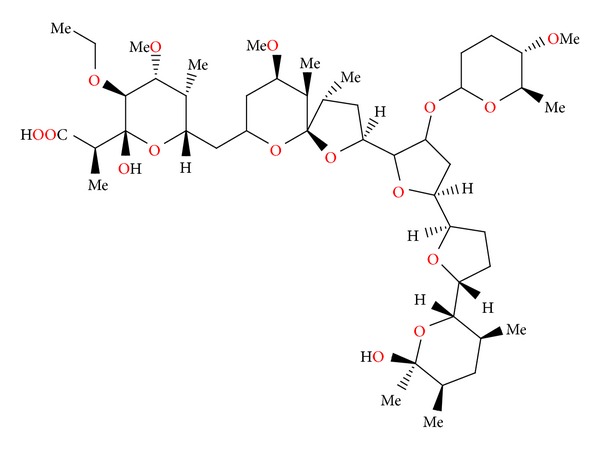
Structure of W341C.

W341C (Figure 62) was isolated from strains of *Streptomyces* W341. This antibiotic is K^+^-selective ionophore that inhibits mitochondrial substrate oxidation. W341C transported K^+^ ion at a greater rate than nigericin, but it transported Na^+^ ion at a lower rate than monensin. W341C is able to induce potassium loss in *Bacillus subtilis* and *Streptococcus lactiae* and promote potassium uptake into *Escherichia coli* [95].

### 2.21. Laidlomycin

**Figure 63:**
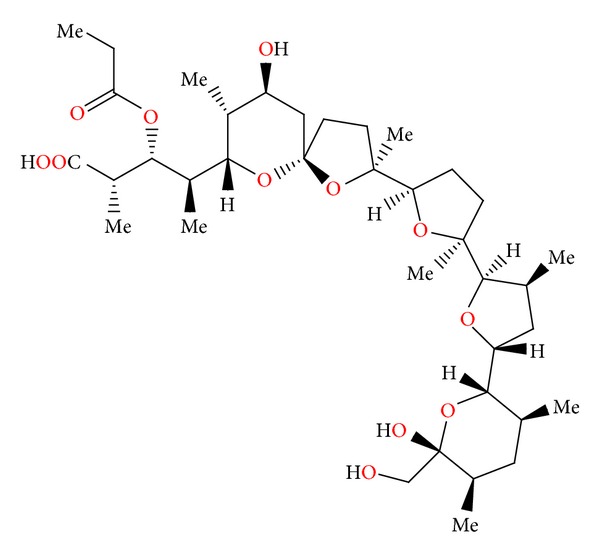
Structure of laidlomycin.

Laidlomycin (Figure 63) was isolated from *Streptomyces eurocidicus*. This antibiotic inhibited growth of some Gram-positive bacteria only at high concentrations such as 50–100 mg/mL, but was not active against Gram-negative bacteria, yeast, and fungi. In broth dilution laidlomycin was active against several *Mycoplasmas*. The acute toxicity of laidlomycin, expressed as LD_50_, was 5 mg/kg (intraperitoneally) and 2.5 mg/kg (subcutaneously) in mice. Antitumor activity against Sarcoma 180 solid tumours in mice and antiviral activity against several viruses *in vitro* were examined but showed no significant effect [96].

### 2.22. CP-84657

**Figure 64:**
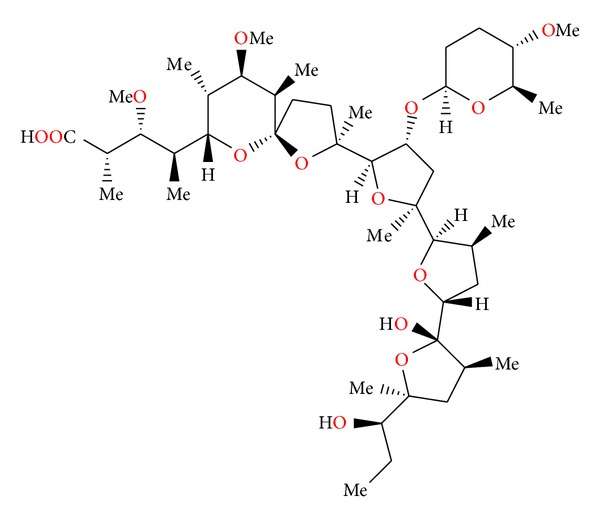
Structure of CP-84657.

CP-84657 (Figure 64) was isolated from *Actinomadura* sp. ATCC 53708. Its crystal structure was determined by X-ray analysis of its rubidium salt. CP-84657 was active against *Eimeria tenella* (major causative agent of chicken coccidiosis) at doses of 5 mg/kg or less in feed. It was also active *in vitro* against certain Gram-positive bacteria, as well as the spirochete *Treponema hyodysenteriae*. No activity was observed against *Escherichia coli* [97].

### 2.23. Grisorixin and Epigrisorixin

**Figure 65:**
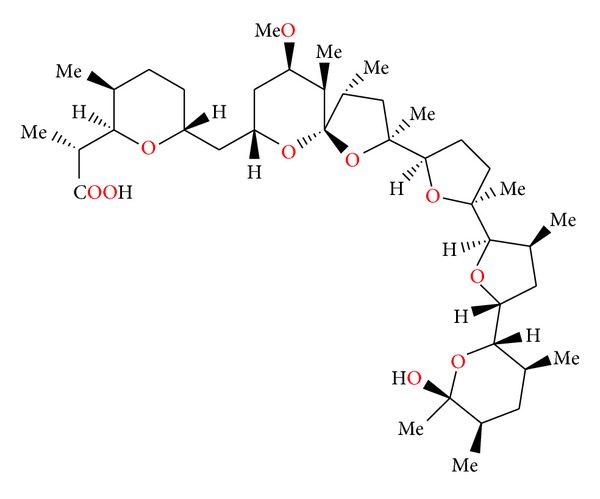
Structure of grisorixin.

 Grisorixin (Figure 65) was isolated from *Streptomyces griseus* [98]. Its crystal structure was determined by X-ray analysis of its thallium and silver salts [99, 100]. Grisorixin showed activity against Gram-positive bacteria, but was shown to be toxic. Determination of the toxicity of grisorixin in mice revealed LD_50_ of 15 mg/kg when the antibiotic was given subcutaneously [98].

**Figure 66:**
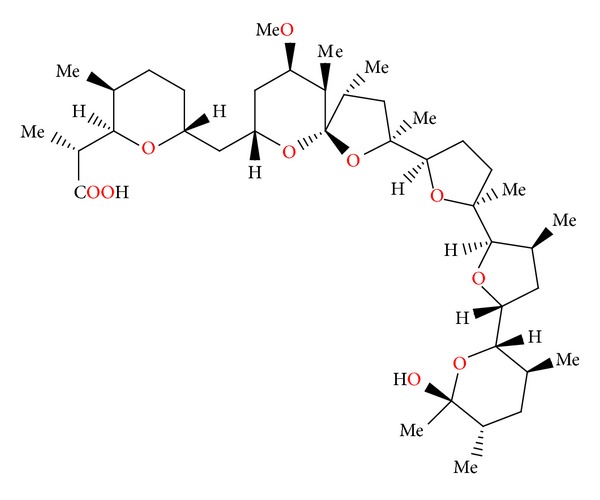
Structure of epigrisorixin.

 Epigrisorixin (Figure 66) was isolated from *Streptomyces hygroscopicus*. Antimicrobial study showed that epigrisorixin is less toxic than grisorixin [101].

### 2.24. CP-54883

**Figure 67:**
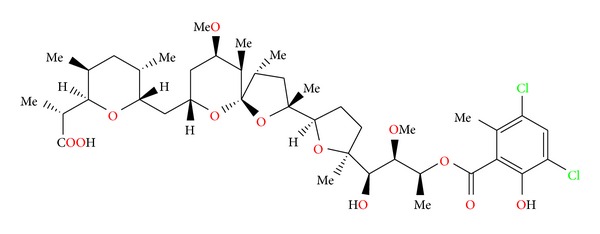
Structure of CP-54883.

CP-54883 (Figure 67) was isolated from *Actinomadura routienii* [102]. Its crystal structure was determined by X-ray analysis of its benzoate derivative [103]. CP-54883 exhibited activity only against Gram-positive bacteria. It was not active against Gram-negative bacteria and yeasts. This antibiotic was active against *Eimeria tenella*, *Eimeria maxima*, and *Eimeria acervulina* coccidian when administrated in feed at 10 to 20 *μ*g/g. Chickens were protected from lesions at the higher levels but suffered from poor weight gains and feed intake [100]. CP-54883 also induced a change in the proportion of volatile fatty acids (acetate, propionate, and butyrate) produced in the rumen by increasing the molar proportion of propionate in the rumen fluids [102].

### 2.25. SF-2487

**Figure 68:**
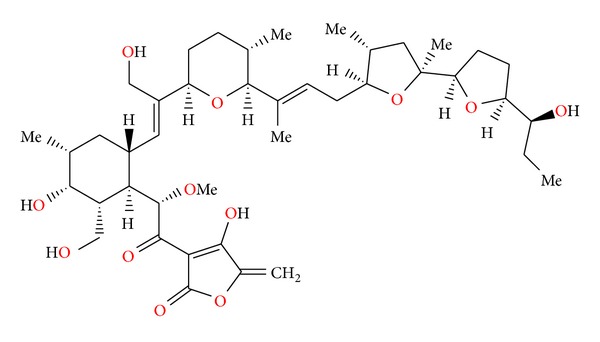
Structure of SF-2487.

SF-2487 (Figure 68) was isolated from a culture broth of *Actinomadura *sp. SF2487. Its crystal structure was determined by X-ray analysis of its silver salt [104]. SF-2487 showed moderate activity against Gram-positive bacteria, but no activity against Gram-negative bacteria. SF-2487 exhibited *in vitro* antiviral activity against influenza virus. The LD_50_ value of SF-2487 was 25 mg/kg by injection in mice [104].

### 2.26. X-14868A, X-14868B, X-14868C, and X-14868D

**Figure 69:**
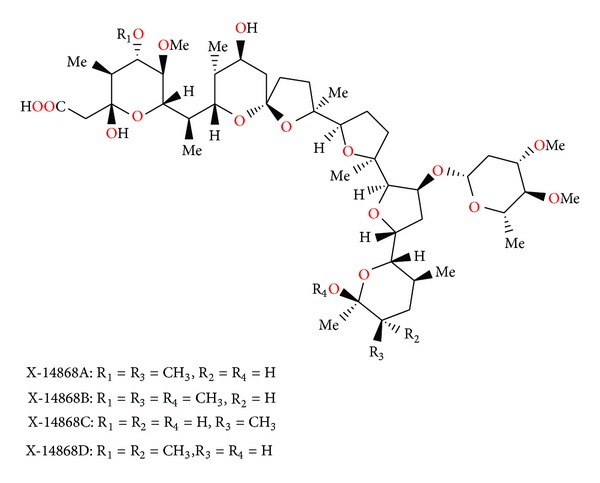
Structures of X-14868A, X-14868B, X-14868C, and X-14868D.

Four polyether antibiotics (Figure 69) were isolated from a culture of *Nocardia* (strain X-14868A, X-14868B, X-14868C, and X-14868D). Crystal structures of each of these antibiotics were determined by X-ray analysis [105]. All four compounds were active against Gram-positive bacteria and exhibited no activity against Gram-negative bacteria and fungi. Antibiotic X-14868A was also active against *Treponema hyodysenteriae* [105].

### 2.27. CP-80219

**Figure 70:**
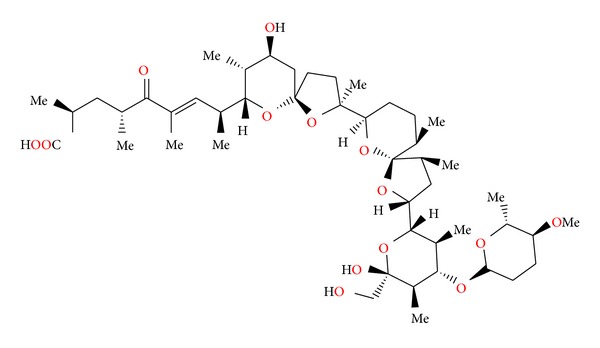
Structure of CP-80219.

 Cp-80219 (Figure 70) was isolated from *Streptomyces hygroscopicus*. Its crystal structure was determined by X-ray analysis of its rubidium salt [106]. CP-80219 sodium salt showed good activity against Gram-positive bacteria as well as the spirochete *Treponema hyodysenteriae*. No activity was observed against Gram-negative bacteria including *Escherichia coli*. It afforded anticoccidial activity between 30 and 120 mg/kg in feed against *Eimeria tenella* in chickens [106].

### 2.28. Moyukamycin

**Figure 71:**
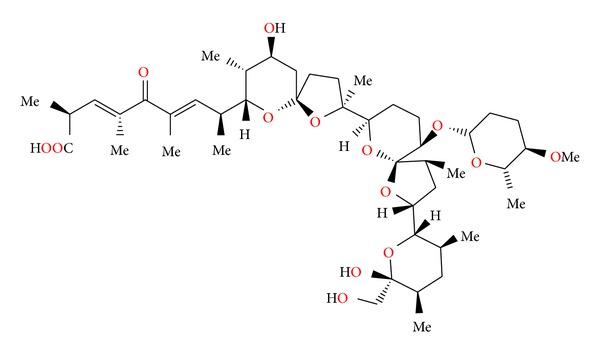
Structure of moyukamycin.

Moyukamycin (Figure 71) was isolated from *Streptomyces hygroscopicus*. It showed activity against a wide range of Gram-positive bacteria, while no activity against Gram-negative bacteria [107].

### 2.29. X-14931A

**Figure 72:**
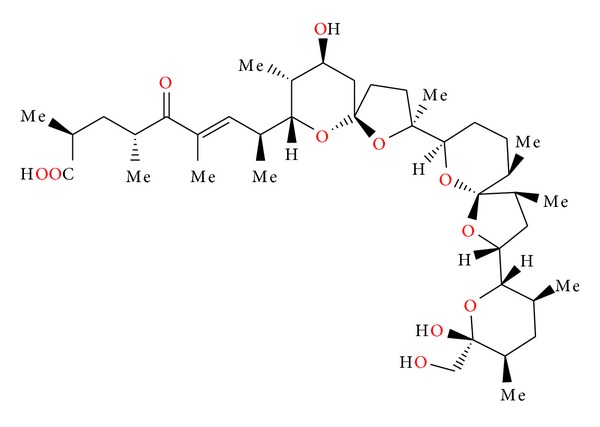
Structure of X-14931A.

X-14931 (Figure 72) was isolated from a culture of *Streptomyces* sp. X-14931. Its crystal structure was determined by X-ray analysis of its silver salt [108]. Antibiotic X-14931A showed *in vitro* activity against Gram-positive microorganisms and yeasts. It was also active against mixed *Eimeria* infection in chickens at 50 *μ*g/g in feed. The antibiotic also exhibited activity in the rumen growth promotant test [108].

### 2.30. X-14873A, X-14873G, and X-14873H

**Figure 73:**
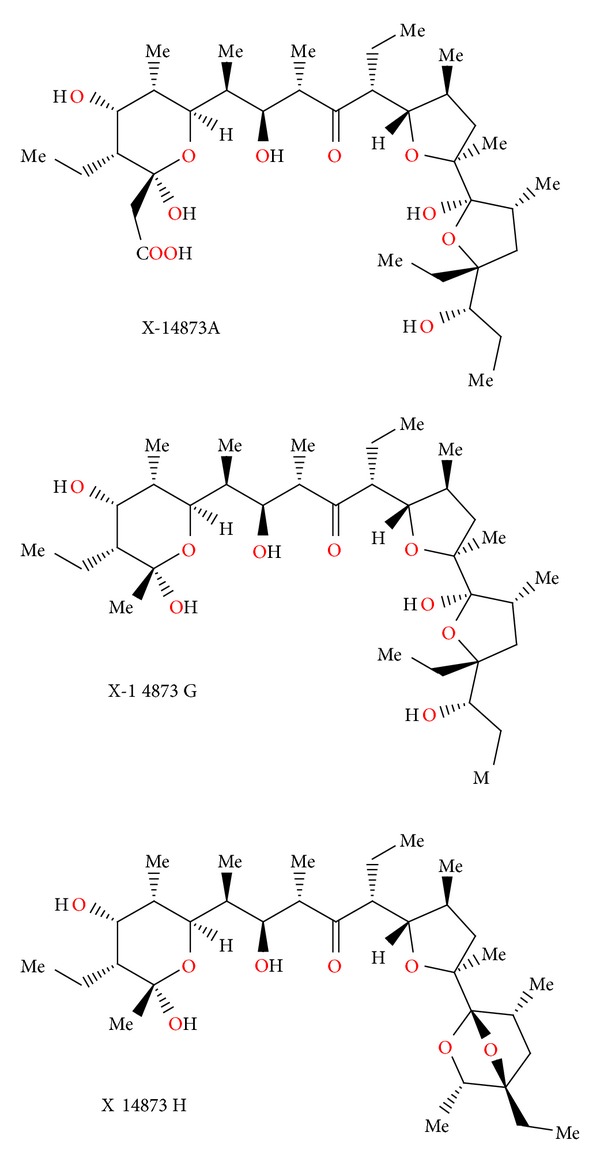
Structures of X-14873A, X-14873G, and X-14873H.

 Three polyether ionophores: X-14873A, X-14873G, and X-14873H (Figure 73) were isolated from the fermentation of *Streptomyces* sp. X-14873 (ATCC31679). Crystal structures of X-14873A and X-14873H were reported [109]. Antibiotic X-14873A was mainly active against Gram-positive bacteria and exhibited no activity against Gram-negative bacteria. It was interesting to note that X-14873H, the descarboxyl derivative of X-14873A, was also active against Gram-positive bacteria, while the other descarboxyl derivative, X-14873G, was practically inactive. These results indicated that the carboxylic function of the polyether antibiotic molecule was not required for the antimicrobial activity, even though the ionized carboxylic group played an important role in complexation of cations by carboxylic acid polyether ionophores [110]. Antibiotic X-14873A also induced a change in the proportion of volatile fatty acids (acetate, propionate, and butyrate) produced in the rumen by increasing the molar proportion of propionate in the rumen fluid [110].

### 2.31. Noboritomycin

**Figure 74:**
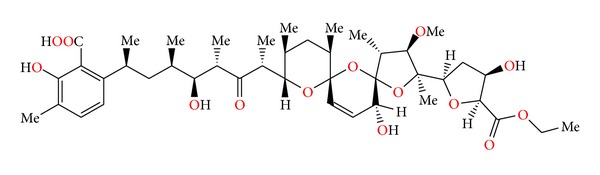
Structure of noboritomycin.

Noboritomycin (Figure 74) was isolated from *Streptomyces noboritoensis*. Crystal structure was determined by X-ray analysis of its silver salt. Noboritomycin was the first polyether ionophore possessing two carboxylic acid functions on the carbon backbone, namely, a free acid and an additional carboxylic acid ethyl ester group [111]. Noboritomycin was active against a wide range of Gram-positive bacteria, but was inactive against Gram-negative bacteria and yeasts. It exhibited only weak anticoccidial activity (*Eimeria tenella*) in chicken [111].

### 2.32.  6-chloronoboritomycin

**Figure 75:**
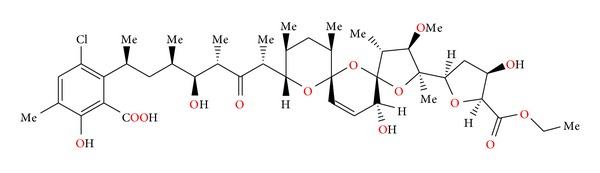
Structure of 6-chloronoboritomycin.

 6-chloronoboritomycin (Figure 75) was isolated from *Streptomyces malachitofuscus* [112]. Its crystal structure was determined by X-ray analysis of its thallium and rubidium salts [113]. This antibiotic is able to complex and transport monovalent as well as divalent metal cations. 6-chloronoboritomycin is active against Gram-positive bacteria and some anaerobes. In addition, it exhibits *in vitro* activity against several strains of *Treponema hyodysenteriae*, a causal agent of swine dysentery [112]. 

### 2.33. CP-82009

**Figure 76:**
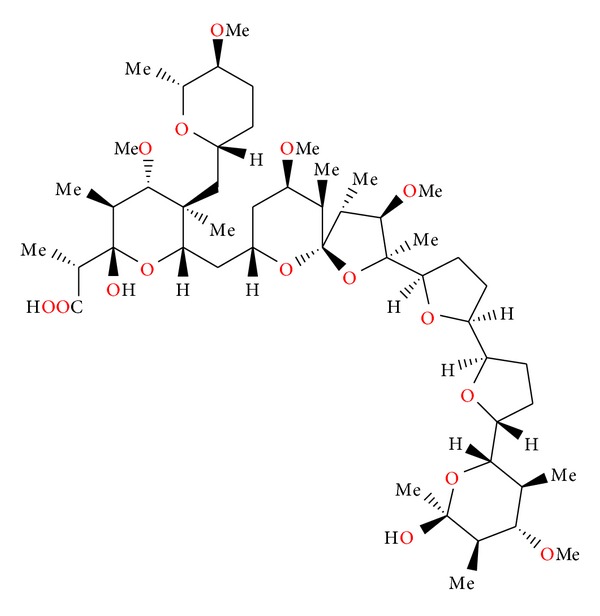
Structure of CP-82009.

 CP-82009 (Figure 76) was isolated by solvent extraction from the fermentation broth of *Actinomadura* sp. ATCC 53676. Its crystal structure was determined by X-ray analysis of its rubidium salt. CP-82009 exhibited activity against Gram-positive bacteria, as well as the spirochete *Treponema hyodysenteriae*. No activity was observed against *Escherichia coli* [114].

### 2.34. Abierixin

**Figure 77:**
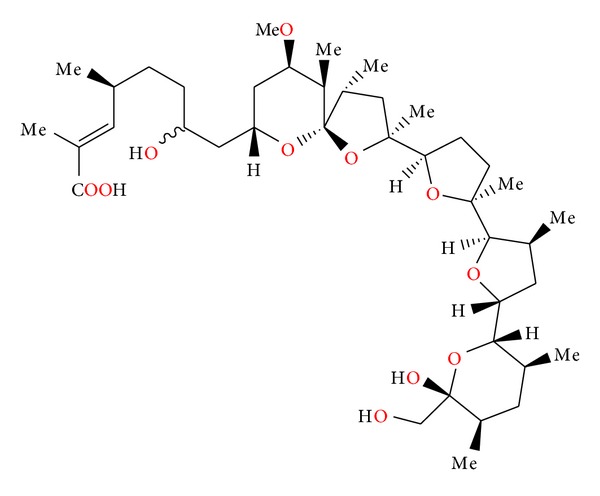
Structure of abierixin.

 Abierixin (Figure 77) was isolated from *Streptomyces albus*. It exhibited weak activity against Gram-positive bacteria. The acute toxicity of abierixin in mice was examined. The LD_50_ value was 80–100 mg/kg. The anticoccidial evaluation of abrexin was carried out with chickens infected with *Eimeria tenella*. Abierixin, at 40 ppm, was effective in reducing the mortality of chickens and in increasing the average body weight of treated infected chickens compared to untreated infected controls [115].

### 2.35. A-83094A

**Figure 78:**
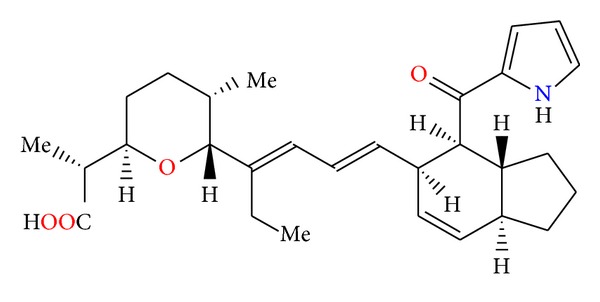
Structure of A-83094A.

 A-83094A (Figure 78) was isolated from the strain of *Streptomyces setonii*. The antibiotic is active against Gram-positive bacteria. At a concentration of 0.31 *μ*g/mL, it completely inhibits the development of *Eimeria tenella*. However, the compound showed no efficacy *in vivo* when chicks infected with *Eimeria tenella* or *Eimeria acervulina* were fed a diet containing 200 *μ*g/g A-83094A [116].

### 2.36. Indanomycin

**Figure 79:**
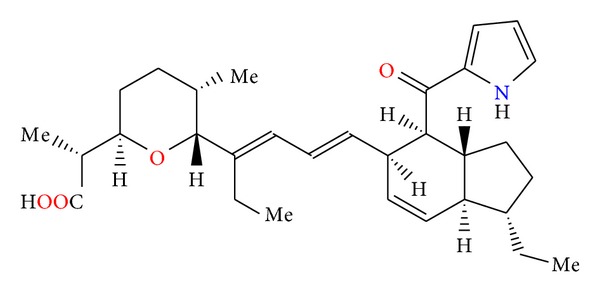
Structure of indanomycin.

Indianomycin (Figure 79) was isolated from the strain of *Streptomyces antibioticus* [117]. Its crystal structure was determined by X-ray analysis of its bromophenethylamine salt [118]. Indianomycin was active *in vitro* against Gram-positive bacteria. It is also effective as a growth promotant for ruminants, increasing feed utilization by these animals [117].

## 3. Conclusion

Polyether ionophores are generally active against Gram-positive bacteria. These compounds are able to form complexes with metal cations and transport them across lipid bilayer. In consequence, the whole process leads to changes in the osmotic pressure inside the cell, causing death of the bacteria cell. Some of them also show anticoccidial activity. Recently, it has been shown that several ionophores exhibit anticancer activities (monensin, salinomycin, and laidlomycin). Results of many studies have shown that both cancer stem cells (CSCs) and multidrug-resistant cancer cells (MDR) are effectively killed by polyether ionophores particularly by salinomycin. Polyether ionophores are currently well-recognized candidates to be clinically tested as anticancer drug candidates. The activity of these compounds should be further studied *in vitro* to specify their mechanisms of action and *in vivo* to assess their activities and tolerance in the different types of cancer. The studies performed so far have shown that these compounds affect cancer cells in a special way by increasing their sensitivity to chemotherapy (monensin, salinomycin, inostamycin) and reverse multidrug resistance (laidlomycin and monensin) in human carcinoma. Furthermore, these compounds have been found to be cytotoxic to the human carcinoma multidrug-resistant cells (monensin and salinomycin). Ionophore antibiotics also inhibit chemoresistant cancer cells by increasing apoptosis, but up to now, only salinomycin has been successfully able to kill human cancer stem cells (CSCs). Therefore, polyether antibiotics should be considered as new anticancer drugs for cancer prevention and cancer therapy, and the mechanism of their anticancer activity should be studied in detail.
